# P2RY8 variants in lupus patients uncover a role for the receptor in immunological tolerance

**DOI:** 10.1084/jem.20211004

**Published:** 2021-12-10

**Authors:** Yuke He, Antonia E. Gallman, Chengmei Xie, Qian Shen, Jianyang Ma, Finn D. Wolfreys, Moriah Sandy, Todor Arsov, Xiaoqian Wu, Yuting Qin, Pingjing Zhang, Simon Jiang, Maurice Stanley, Philip Wu, Jingjing Tan, Huihua Ding, Haiyan Xue, Wei Chen, Jinping Xu, Lindsey A. Criswell, Joanne Nititham, Marcin Adamski, A. Richard Kitching, Matthew C. Cook, Lanfang Cao, Nan Shen, Jason G. Cyster, Carola G. Vinuesa

**Affiliations:** 1 Centre for Personalised Immunology, Renji Hospital, School of Medicine, Shanghai Jiao Tong University, Shanghai, China; 2 Howard Hughes Medical Institute and Department of Microbiology and Immunology, University of California, San Francisco, San Francisco, CA; 3 Centre for Personalised Immunology, John Curtin School of Medical Research, Australian National University, Australian Capital Territory, Australia; 4 Shanghai Institute of Rheumatology, Renji Hospital, School of Medicine, Shanghai Jiao Tong University, Shanghai, China; 5 Department of Pediatrics, Renji Hospital, Shanghai Jiao Tong University, Shanghai, China; 6 Russell/Engleman Rheumatology Research Center, Department of Medicine, University of California, San Francisco, San Francisco, CA; 7 Centre for Personalised Immunology, Centre for Inflammatory Diseases, Monash University Department of Medicine, Clayton, Victoria, Australia; 8 Francis Crick Institute, London, UK

## Abstract

B cell self-tolerance is maintained through multiple checkpoints, including restraints on intracellular signaling and cell trafficking. P2RY8 is a receptor with established roles in germinal center (GC) B cell migration inhibition and growth regulation. Somatic *P2RY8* variants are common in GC-derived B cell lymphomas. Here, we identify germline novel or rare *P2RY8* missense variants in lupus kindreds or the related antiphospholipid syndrome, including a “de novo” variant in a child with severe nephritis. All variants decreased protein expression, F-actin abundance, and GPCR-RhoA signaling, and those with stronger effects increased AKT and ERK activity and cell migration. Remarkably, P2RY8 was reduced in B cell subsets from some SLE patients lacking *P2RY8* gene variants. Low P2RY8 correlated with lupus nephritis and increased age-associated B cells and plasma cells. By contrast, P2RY8 overexpression in cells and mice restrained plasma cell development and reinforced negative selection of DNA-reactive developing B cells. These findings uncover a role of P2RY8 in immunological tolerance and lupus pathogenesis.

## Teaser

Somatic mutations in P2RY8 that promote B cell growth and migration are common in lymphomas. He et al. describe germline loss-of-function *P2RY8* variants in SLE and uncover novel functions of P2RY8 in immunological tolerance through restraining plasma cell development and promoting B cell–negative selection.

## Graphical Abstract

**Figure:**
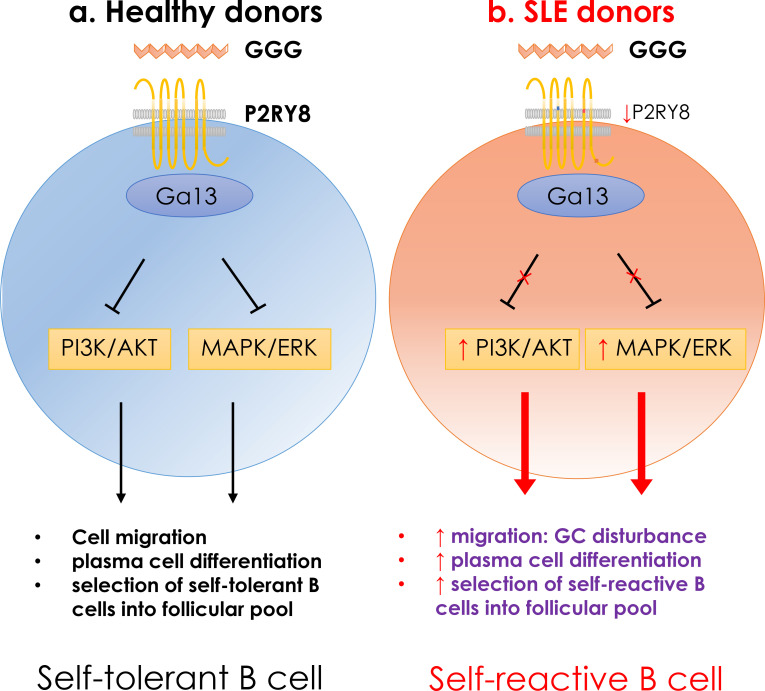

## Introduction

P2RY8 is a Gα13 protein-coupled receptor that is found in humans, with orthologues in many other vertebrates but not rodents (Muppidi et al., 2014). It is highly expressed in germinal center (GC) B cells. S-geranylgeranyl-L-glutathione (GGG) is a potent ligand for P2RY8, which inhibits B cell AKT activation and chemokine-directed migration (Lu et al., 2019). Somatic mutations in both *P2RY8* and *GNA13* (the gene encoding Gα13) have been reported in many GC-derived B cell lymphomas (Lohr et al., 2012; Muppidi et al., 2014). These studies have established an important role for P2RY8 in GC B cell confinement and growth regulation. However, P2RY8 is expressed widely by human lymphocytes (Lu et al., 2019; Mullighan et al., 2009; Wu et al., 2013), suggesting broader roles in the immune system.

The genetic contribution to systemic lupus erythematosus (SLE) is substantial, but inheritance is seldom Mendelian (Alarcón-Segovia et al., 2005; Hunt et al., 2013). Genome-wide association studies have revealed that multiple common variants confer genetic susceptibility to SLE, defining key pathways involved in disease pathogenesis (Visscher et al., 2017). There is nevertheless increasing evidence that “de novo,” novel, and rare gene variants with strong effects can also cause or contribute significantly to disease, particularly in patients with early-onset lupus (Ghodke-Puranik and Niewold, 2015; Jiang et al., 2019; Lo, 2016; Marouli et al., 2017; Omarjee et al., 2019). In accord with the delicate balances associated with maintenance of immunological tolerance, in a number of cases heterozygosity for loss-of-function variants has been associated with disease (Almlöf et al., 2019; Crow, 2013; Lo, 2016).

Here, we describe the first novel, ultrarare (minor allele frequency [MAF] < 0.0001) and rare (MAF < 0.005) germline *P2RY8* variants, identified in patients with SLE or lupus-related antiphospholipid syndrome (APS). Our allelic series reveals a spectrum of severity in the functional effects, with a de novo variant conferring a complete loss of function, an ultra-rare variant behaving as a hypomorphic allele, and a less-rare variant only conferring subtle defects. These functional defects included a reduced ability to repress AKT activation and migration, and we also found deregulated ERK activation. Our work provides evidence for novel functions of P2RY8 in restraining plasma cell development and limiting the selection of DNA-reactive B cells into the follicular pool. Loss of P2RY8 is likely to disrupt B cell tolerance and contribute to lupus pathogenesis.

## Results

### Germline *P2RY8* variants in autoimmune patients

**Figure 1.**
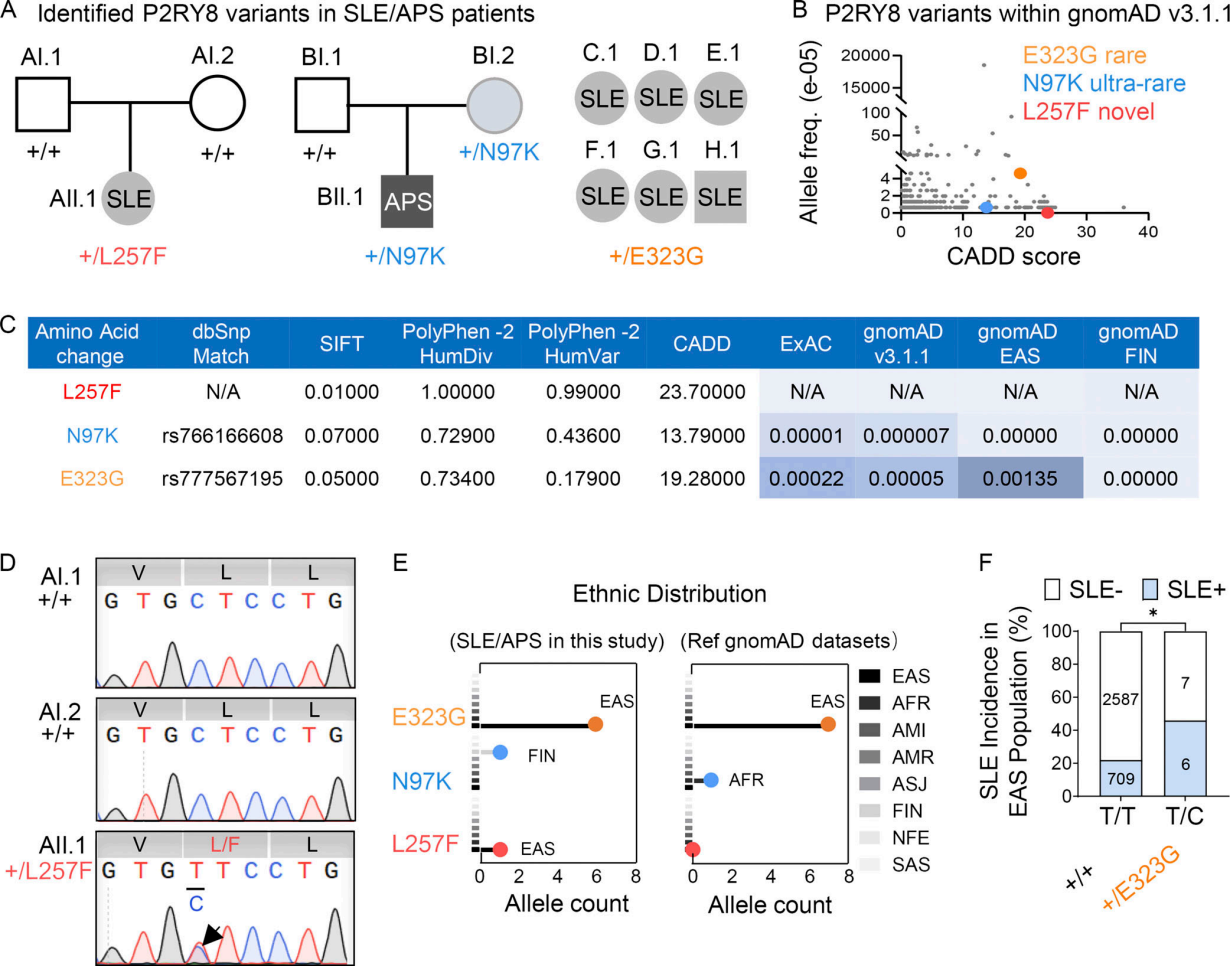
**Identification of P2RY8 variants in patients with SLE. (A)** Left and center: Pedigrees showing affected probands and parents. Right: Summary of six affected probands. **(B)** P2RY8 variants presented in reference gnomAD v3.1 database (http://gnomad.broadinstitute.org). **(C)** Deleterious significance of each variant was predicted based on three tools, including PolyPhen-2, SIFT, and CADD. The ExAC, gnomAD allele frequency, and SNP database (dbSNP) match shown as indicated. HumDiv/HumVar, PolyPhen-2 classifiers for evaluating missense mutations; FIN, Finnish. **(D)** Sanger confirmation of P2RY8 L257F variant. V, valine; L, leucine; L/F, leucine/phenylalanine. **(E)** Population distribution of P2RY8 variants in affected probands or in reference (ref) gnomAD v3.1 datasets. Line colors correspond to ethnic group. AFR, African/African American; AMI, Amish; AMR, American Admixed/Latino; ASJ, Ashkenazi Jewish; NFE, Non-Finnish European; SAS, South Asian. Total alleles in EAS marked in bold. **(F)** Graph showing SLE incidence in *+/+* versus *+/E323G* EAS population. *n* = 715, EAS SLE analyzed in this study (blue); *n* = 2,594, EAS control donors in reference gnomAD v3.1 database (white). P = 0.0312 (χ^2^ test), P = 0.0423 (Fisher’s exact test). *, P < 0.05. freq., frequency.

**Figure S1.**
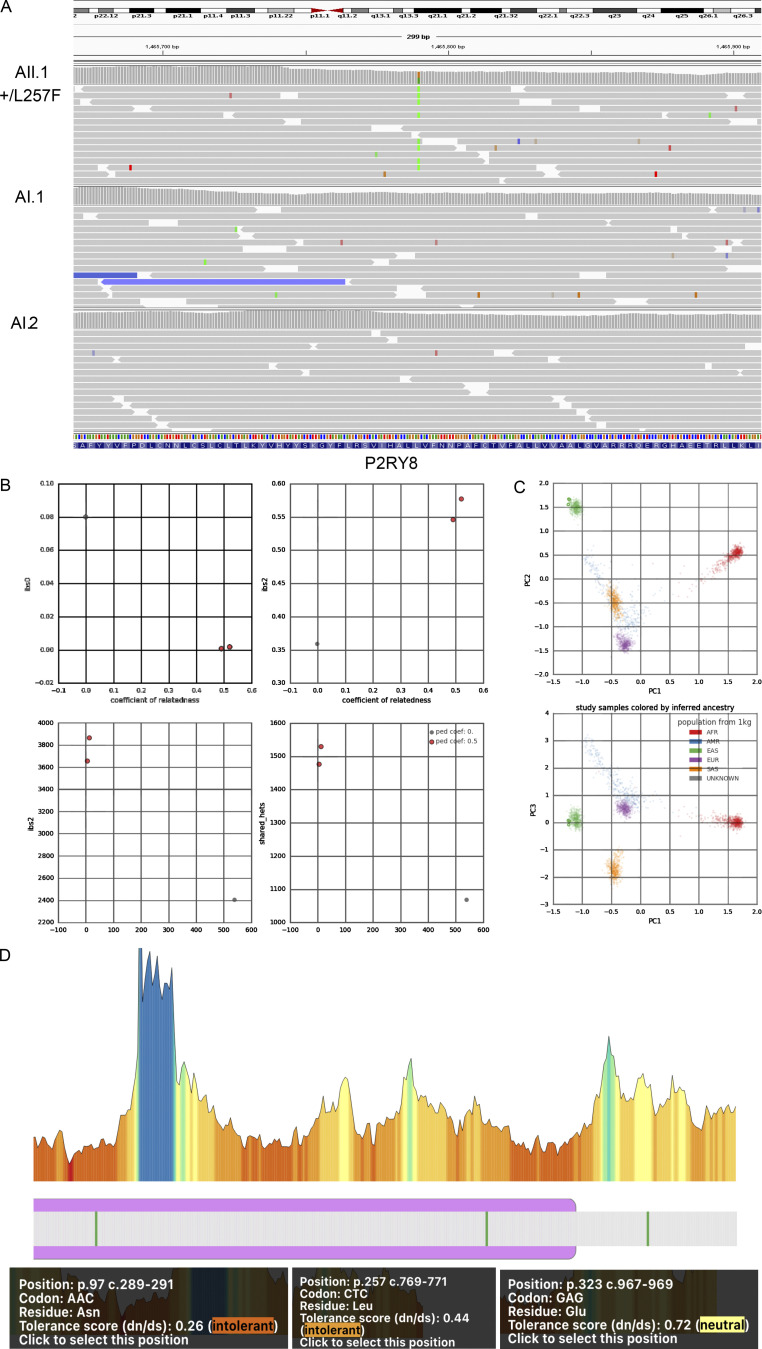
**Identification of L257F variant in a patient with SLE. (A)** P2RY8 +/L257F variant viewed by Integrative Genomics Viewer. The green bars represent bases Ts that did not match the reference bases Cs. **(B and C)** Familial-relatedness (B) and ancestry (C) check using Peddy. Ped Coef, Peddy relatedness coefficient. Coefficients of 0.5 (parents-offspring or full siblings) and 0 (unrelated). **(D)** Illustration of tolerance of missense variants in *P2RY8* gene (Wiel et al., 2019). AFR, African/African American; AMR, American Admixed/Latino; EUR, European; SAS, South Asian.

We performed whole-exome sequencing of 61 Chinese trios in which the probands had childhood-onset SLE and identified a de novo heterozygous missense *P2RY8* variant (p.Leu257Phe) (c.769C>T; Fig. 1 A and Fig. S1 A) in a child born from nonconsanguineous parents with no significant family history. Databases of human genetic variation, including Genome Aggregation Database (gnomAD; Fig. 1 B) and Exome Aggregation Consortium (ExAC) indicated the L257F variant was novel and was predicted to be damaging according to Polymorphism Phenotyping (PolyPhen), Sorting Intolerant from Tolerant (SIFT), and Combined Annotation-Dependent Depletion (CADD; Fig. 1 C). Sanger sequencing (Fig. 1 D) and Peddy’s relatedness test (Fig. S1, B and C) confirmed paternity (Pedersen and Quinlan, 2017) and that the L257F variant had arisen de novo. Serology revealed hypocomplementemia (C3, C4, and CH50), high-titer antinuclear antibodies (ANAs; 1:1,280), and anti–double-stranded DNA (dsDNA), anti-Smith (anti-Sm), anti-U1RNP, anti-Ro/SSA, and thyroglobulin autoantibodies. Urine analysis revealed proteinuria (max 1,629.3 mg/24 h) and hematuria. Renal biopsy showed class V membranous lupus nephritis with abundant IgG, IgA, and light Ig chain deposits and moderate granular deposits of C1q and C3. She was treated with methylprednisolone, hydroxychloroquine (HCQ), and mycophenolate mofetil (MMF).

Analysis of whole-exome sequencing from over 2,000 Australian (AU) subjects enriched in kindreds with systemic autoimmunity identified a proband of Finnish European ethnicity, with APS carrying an ultrarare (MAF = 0.000007; 1/152,206 alleles recorded in gnomAD) *P2RY8* variant: N97K (c.291C>G; Fig. 1, A–C). He was diagnosed after presenting at age 45 with a chronic, nonhealing, left lateral ankle ulcer and digital ischemia complicated by possible osteomyelitis, leading to amputation of the first and second toes. The histology was consistent with thrombotic vasculopathy, which occurred despite anticoagulation that had been prescribed when he was noted to be in atrial fibrillation 10 yr earlier. Past medical history also included dilated cardiomyopathy and mild congenital intellectual development. Additional investigations revealed very high levels of antiphospholipid antibodies, with anti-cardiolipin IgG >2,024 chemitluminescent units (CU; reference range, <20) and anti–β2 tglycoprotein I IgG >6,100 CU (reference range, <20; testing for tlupus anticoagulant was precluded by concurrent antitcoagulation). He also had a low ANA titer (1:80, nucleolar pattern; reference range, <1:40). The allele was inherited from his mother, who had a high titer of autoantibodies to SSA 52 kD at 492 CU (reference range, <20), and a family history of recurrent miscarriages, a common finding in APS.

Sequencing of additional SLE cases from cohorts in China, Australia, and the United States also identified another rare heterozygous *P2RY8* variant, E323G (c.968A>G), in six unrelated SLE patients, all of Chinese ethnicity (Fig. 1, A and E). According to gnomAD, this variant has not been found in any population other than East Asians (EASs; Fig. 1 E), with only seven alleles reported (EAS MAF = 0.0013; Fig. 1, C and E). The variant was deleterious according to SIFT and CADD (Fig. 1 C). Moreover, EAS individuals carrying the variant allele A/G (T/C on the forward strand) exhibited higher incidence of SLE (Fig. 1 F) based on a χ^2^ test of our 715 sequenced EAS SLE individuals and 2,594 EAS control individuals in gnomAD v3.1.1 datasets. All patients with the E323G variant were positive for SSA/SSB/dsDNA/nucleosome/histone and other lupus autoantibodies, and two of six had lupus nephritis. No other rare *P2RY8* variants were identified in these cohorts.

### Characterization of P2RY8 variants indicates loss of function

**Figure 2.**
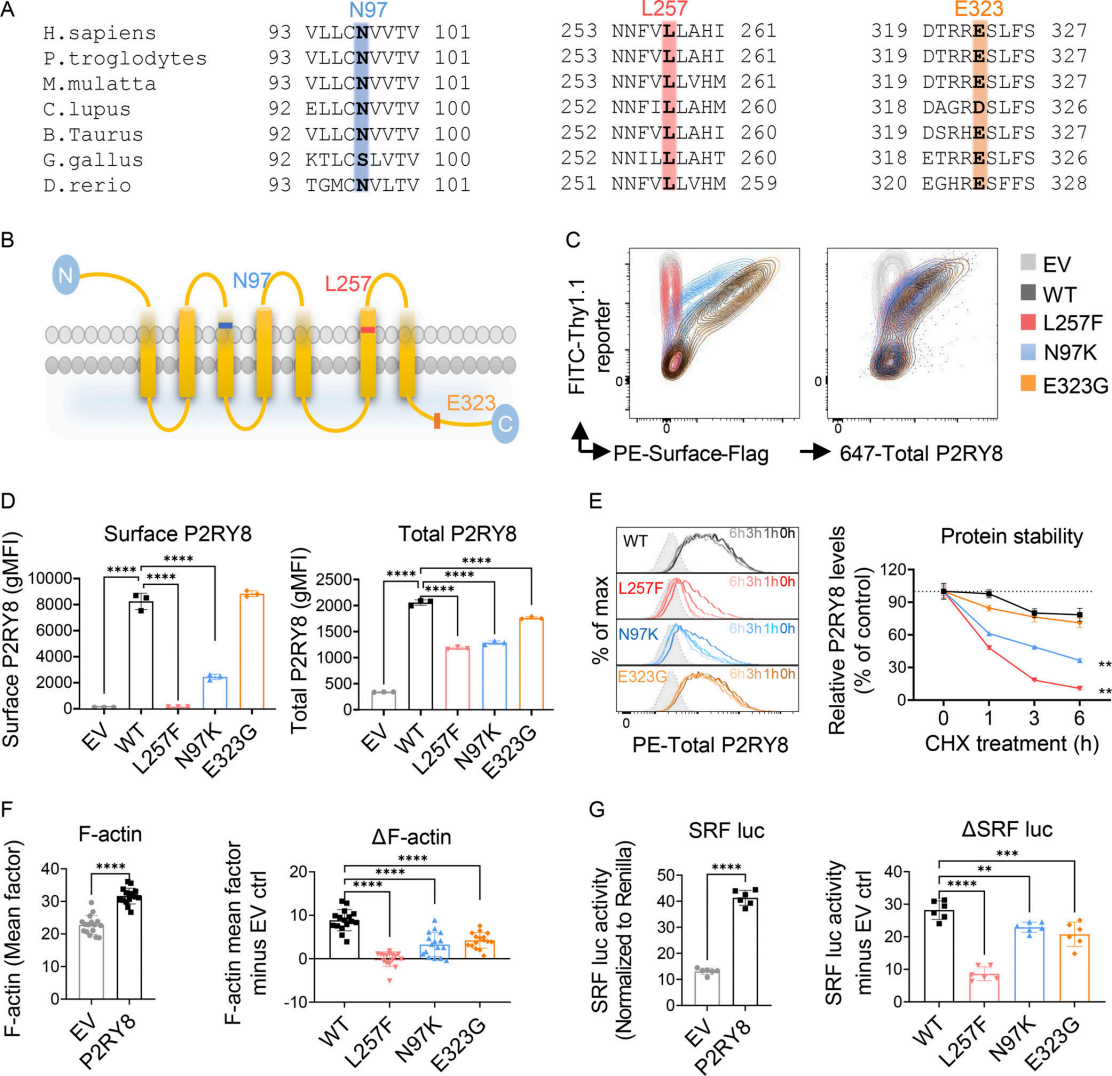
**Characterization of P2RY8 variants indicate loss of function. (A)** Evolutionary conservation of L257 (red), N97 (blue), and E323 (orange) in P2RY8. **(B)** Location of variants in the domains of human P2RY8. **(C and D)** NIH/3T3 cells were transduced with retroviruses encoding FLAG-tagged–WT-P2RY8–IRES-Thy1.1 or mutants. Representative FACS plots (C) and summary graphs (D) showing P2RY8 expression. Surface staining of P2RY8 with anti-FLAG (*n* = 3 for each group). Total P2RY8 staining with anti-P2RY8, which targets the cytoplasmic portion of P2RY8 (*n* = 3 for each group). EV, empty vector. **(E)** Degradation assay of WT or mutant P2RY8 proteins. Representative histograms and summary graphs showing P2RY8 expression after CHX treatment. Leftmost gray histogram (dotted line) depicts background staining in empty vector–transduced cells. Values are relative to time 0 and are means of three replicates. **(F)** Immunofluorescence analysis of F-actin expression. Basic intensity quantification was performed for each image. *n* = 16 images for each group. ΔF-actin was calculated as F-actin mean factor minus EV control (EV ctrl). **(G)** SRF luciferase (SRF luc) assay in HEK293 cells transfected with P2RY8-WT or mutants. (*n* = 6 for each group). ΔSRF activity was calculated as activity minus EV ctrl. Data are representative of three (C, D, and G), four (E), or six (F) independent experiments. P values determined by one-way ANOVA (D, F-right, and G-right), two-way ANOVA with Dunnett's multiple comparisons test (E), or unpaired *t* test (F-left and G-left). **, P < 0.01; ***, P < 0.001; ****, P < 0.0001. Graphs depict mean with SD. gMFI, geometric mean; max, maximum; PE, phycoerythrin.

Consistent with their high CADD scores, the novel L257F and ultrarare N97K variants occur in highly conserved transmembrane regions (Fig. 2, A and B) that are predicted to be intolerant to substitution according to MetaDome analysis (Fig. S1 D; Wiel et al., 2019). The less-rare E323G variant is located in the C-terminus, and this residue is also conserved in multiple species (Fig. 2, A and B). Although 71 additional rare (MAF < 0.005) alleles with CADD scores >12 were present in gnomAD v3.1.1 that contains data for 76,156 individuals sequenced, only four of them are predicted to be loss of function, and two of these with low confidence. Additionally, in the gnomAD v3.1.1 control dataset, which contains only samples collected specifically as controls for disease studies or samples belonging to biobanks, there was only one P2RY8 predicted loss-of-function variant identified among 16,465 individuals sequenced. This is a Glu356 truncation, which is unlikely to be disruptive given that it removes only the last three amino acids.

We next examined the effects of *P2RY8* variants on protein function. Cells of the murine fibroblast line, NIH/3T3, were transduced with Flag-tagged WT or mutant P2RY8 or empty vector, together with an IRES-Thy1.1 reporter. Flow cytometric analysis with an anti-Flag antibody showed a lack of surface expression of the L257F variant and reduced surface expression of the N97K variant (Fig. 2, C and D). Staining with an antibody against the C-terminal cytoplasmic domain showed that the L257F variant conferred a substantial reduction in total P2RY8 protein expression. A similar, although less severe, phenotype was conferred by N97K. E323G resulted in a small, albeit statistically significant, reduction in total P2RY8 expression and no change in surface expression (Fig. 2, C and D). Addition of cycloheximide (CHX) to inhibit protein translation revealed that the transmembrane L257F and N97K mutants of P2RY8 underwent faster degradation, indicating loss of protein stability (Fig. 2 E).

**Figure S2.**
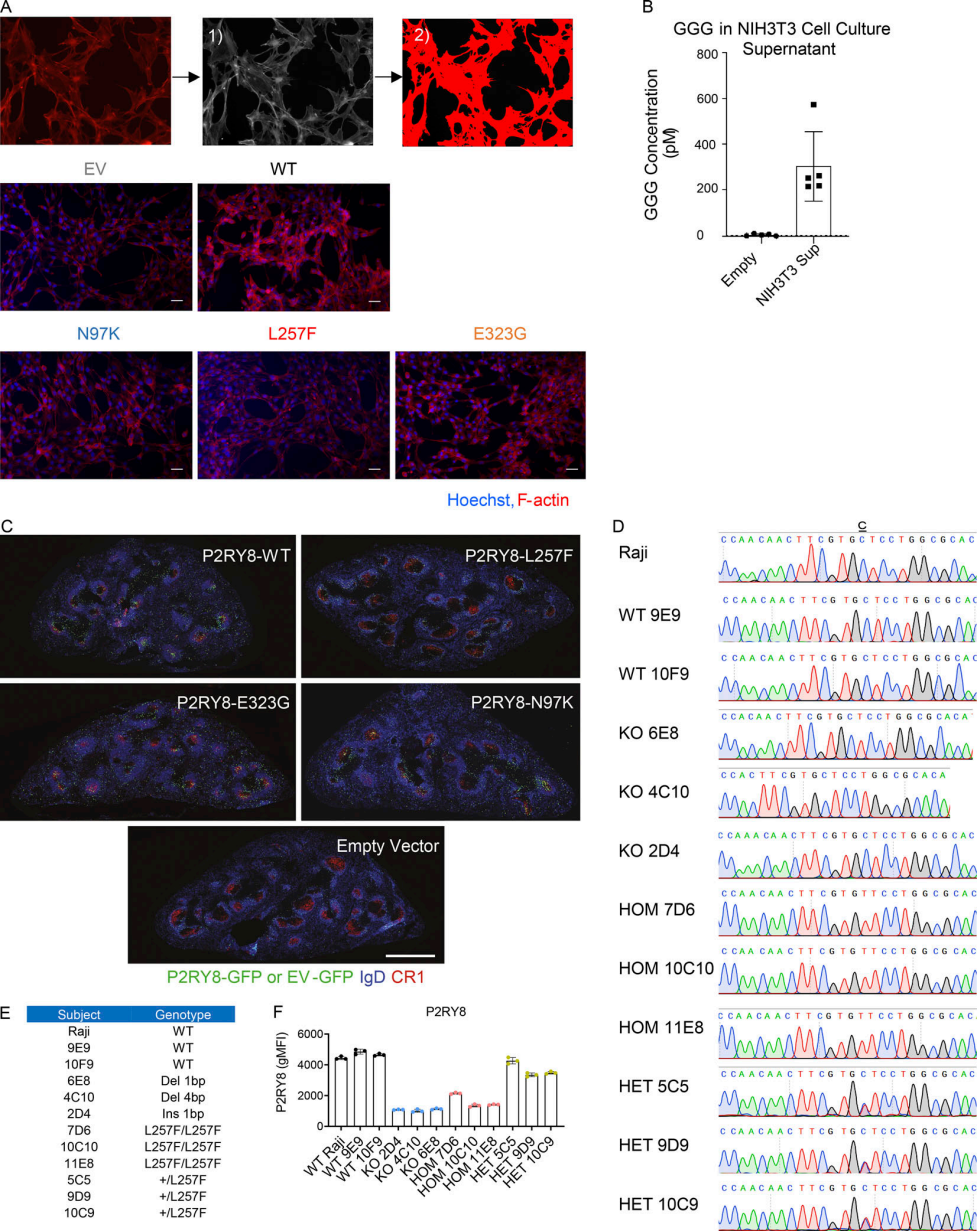
**Characterization of P2RY8 variants indicate loss of function. (A)** Representative images of immunofluorescence staining of F-actin (red). Nuclei were stained with Hoechst 33342 (blue). Scale bar is 50 µm. Quantification was performed as (1) convert single-channel color images to grayscale before proceeding; (2) adjust the threshold, limit the measured area to just the object (red), and measure the intensity. Data were representative of six independent experiments. **(B)** NIH/3T3 cells were seeded at density of 5 × 10^4^/well in 500 µl DMEM cell culture medium supplemented with 10% FBS and 1% penicillin/streptomycin. Cells were cultured for 5 d without replacing culture medium, and GGG levels were measured in the culture supernatant (Sup) using LC-MS/MS (*n* = 5). Empty refers to culture medium incubated in neighboring wells with no cells (*n* = 3). **(C)** Purified splenic B cells were transduced with retroviruses encoding P2RY8-WT-GFP or mutants. Immunofluorescence for P2RY8 or empty vector (EV)–transduced B cells (GFP, green) in the germinal centers (CR1, red) of SRBC immunized mice relative to endogenous follicular B cells (IgD, blue). Scale bar is 500 µm. **(D)** Sanger sequencing confirmation of Cas9-mediated genomic editing in Raji B cell line. **(E)** Summary graph showing genotypes of each CRISPR cell line. **(F)** Flow cytometry analysis of P2RY8 expression in Raji CRISPR cell lines (*n* = 3 replicates for each group). Representative of two independent experiments. Graphs depict mean with SD. gMFI, geometric mean; HOM, homogeneous; HET, heterogeneous.

Gα13 signaling has been reported to promote F-actin formation (Girkontaite et al., 2001; Healy et al., 2016), and consistent with this, we observed that P2RY8 increased the abundance of F-actin in NIH/3T3 cells (Fig. 2 F and Fig. S2 A). Culture supernatants of NIH/3T3 cells contained GGG, the ligand of P2RY8, at amounts sufficient to activate the receptor (Fig. S2 B; Lu et al., 2019). In contrast to the effect of WT P2RY8 on F-actin abundance, cells expressing any of the three P2RY8 variants did not increase F-actin or increased it to a lesser extent, suggesting decreased signaling ability (Fig. 2 F). Serum response factor (SRF) response element luciferase assays read out G protein-coupled receptors–RhoA signaling (Cheng et al., 2010). All three P2RY8 mutants significantly reduced SRF luciferase activity relative to WT P2RY8, again indicating loss of function (Fig. 2 G).

### P2RY8 L257F and N97K variants show reduced inhibition of AKT and ERK

**Figure 3.**
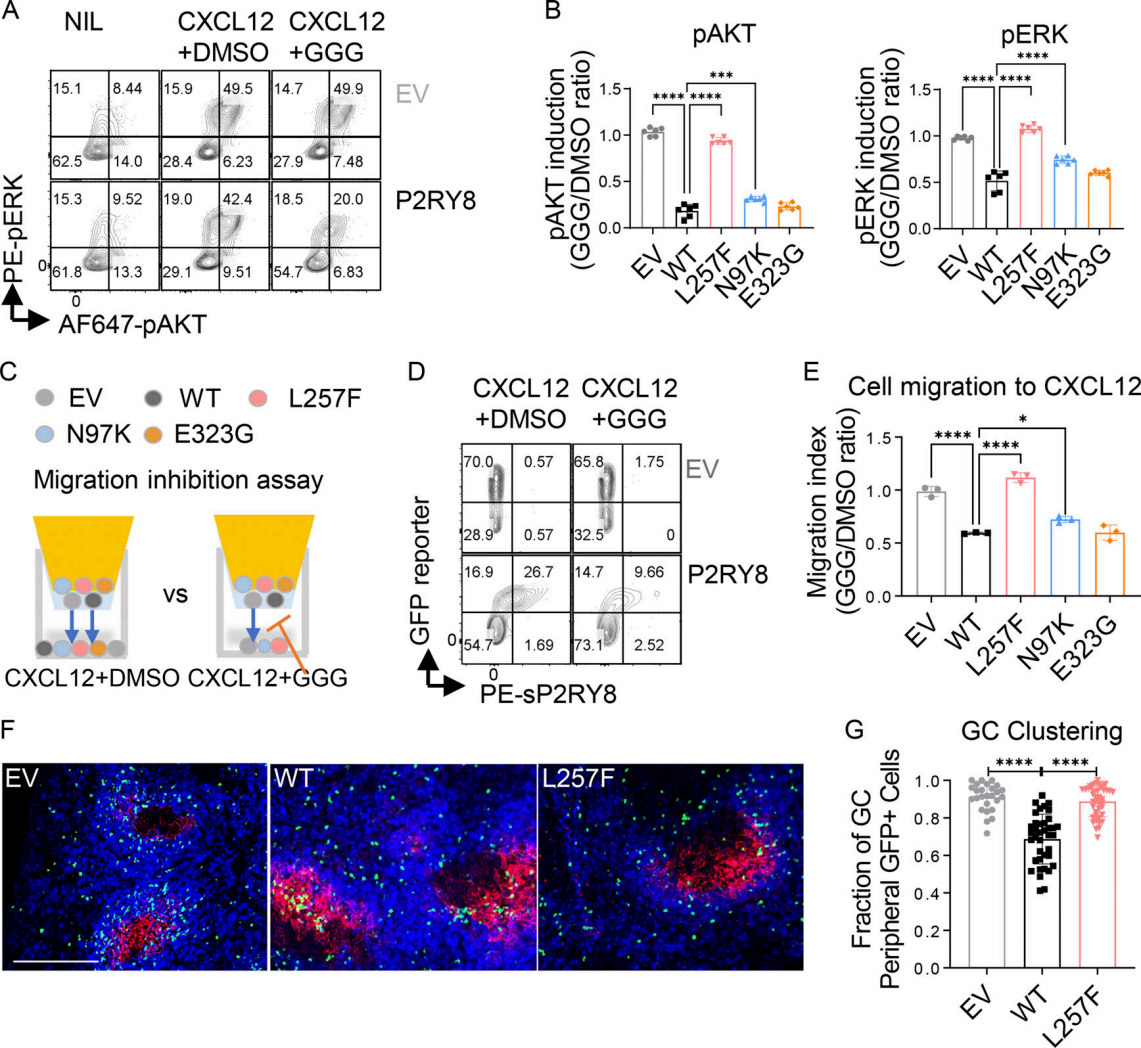
**Functional validation of P2RY8 variants in retrovirally transduced B cells.** Purified splenic B cells were preactivated and transduced with retroviruses encoding P2RY8-IRES-GFP or mutants. **(A and B)** Transduced B cells were treated with either CXCL12+carrier (DMSO) or CXCL12+GGG. **(A)** Representative plots showing pAKT or pERK expression after treatments. **(B)** pAKT or pERK induction index was calculated as phosphorylation induced by CXCL12+GGG divided by mean value of phosphorylation induced by CXCL12+DMSO (*n* = 6 for each group). **(C–E)** Transwell assay of transduced B cells toward CXCL12+DMSO or CXCL12+50 nM GGG. Blue arrows indicate the direction of cell migration. Representative plots (D) and quantification (E) showing migrated cells of the indicated types. Migration index was calculated as cell migration to CXCL12+GGG divided by cell migration to CXCL12+DMSO (*n* = 3 for each group). Data in E is also representative of three experiments in transduced WEHI231 cells. **(F and G)** GC clustering behavior in transduced B cells. Immunofluorescence for P2RY8- or EV-transduced B cells (GFP, green) in the GCs (CR1, red) of SRBC immunized mice relative to endogenous follicular B cells (IgD, blue). Scale bar, 200 µm. **(G)** Quantification of GFP^+^ cells in the outer region of a B cell follicle as a fraction of total GFP^+^ cells in a follicle. Each point represents a B cell follicle, and all follicles of a defined size or larger were quantified from three spleen sections per mouse (*n* = 26 EV, 38 WT, 41 L257F). Data representative of two (F and G) or at least five (A–E) independent experiments. P values determined by one-way ANOVA with Dunnett's multiple comparisons (B, E, and G). *, P < 0.05; ***, P < 0.001; ****, P < 0.0001. Graphs depict mean with SD. EV, empty vector; NIL, non-treated control; PE, phycoerythrin.

P2RY8 has been previously shown to inhibit AKT activation in a GGG-dependent manner (Lu et al., 2019). Since we identified the *P2RY8* variants in patients with systemic autoimmunity, we next tested the hypothesis that these variants interfered with GGG-mediated inhibition of B cell activation. LPS-preactivated mouse B cells were retrovirally transduced with Flag-tagged WT or mutant P2RY8 or empty vector as a control, all containing an IRES-GFP reporter. While P2RY8 is not expressed in rodents, GGG production is conserved, making mice an excellent system in which to evaluate the function of human *P2RY8* alleles (Lu et al., 2019; Muppidi et al., 2015; Muppidi et al., 2014). Moreover, the approach of introducing human genes into mice has been informative in many contexts where a mouse orthologue is lacking (Asfaha et al., 2013; Barrow et al., 2018; Bournazos et al., 2020; Eckhardt and Bastian, 2021; Launay et al., 2000). Phospho-flow cytometric results showed that GGG suppressed CXCL12-induced AKT phosphorylation (pAKT) in B cells expressing P2RY8-WT but not P2RY8-L257F. Suppression was also impaired by the N97K variant, albeit to a lesser extent (Fig. 3, A and B). MAPK/ERK also mediates lymphocyte activation, proliferation, and differentiation, and Gα13 signaling can inhibit ERK (Voyno-Yasenetskaya et al., 1996). As seen with AKT, GGG inhibited CXCL12-mediated ERK phosphorylation (pERK), and P2RY8 L257F and N97K variants interfered with this function (Fig. 3, A and B). The less-rare E323G variant did not affect GGG-dependent AKT or ERK inhibition.

### P2RY8-L257F fails to mediate migration inhibition

P2RY8 has a well-established ability to inhibit B cell migration in response to chemokines (Lu et al., 2019). We next used a transwell assay to assess chemokine (CXCL12)-induced B cell migration in vitro (Fig. 3 C). The administration of GGG inhibited ∼50% of B cells expressing WT P2RY8 from migrating toward CXCL12. By contrast, inhibition of cell migration by GGG was impaired or absent in P2RY8 N97K– and L257F-expressing B cells, respectively (Fig. 3, D and E). Although mice lack a P2RY8 orthologue, retrovirally transduced P2RY8 is sufficient to position adoptively transferred activated mouse B cells within preformed GCs of recipient mice (Lu et al., 2019; Muppidi et al., 2014). To test whether P2RY8 variants disrupted B cell confinement in GCs, B cells were retrovirally transduced to express P2RY8 then transferred to preimmunized recipients, and their location was examined a day later. While WT P2RY8 promoted B cell confinement to GCs, the L257F variant did not (Fig. 3, F and G; and Fig. S2 C). The N97K and E323G variants did not interfere with confinement (Fig. S2 C). However, it should be kept in mind that this assay in mice involves overexpression of the receptor (P2RY8), and it remains possible that the reduced ability to inhibit cell migration observed for N97K in vitro (Fig. 3 E) contributes to the human in vivo phenotype.

### Heterozygosity for P2RY8-L257F allele leads to P2RY8 haploinsufficiency

**Figure 4.**
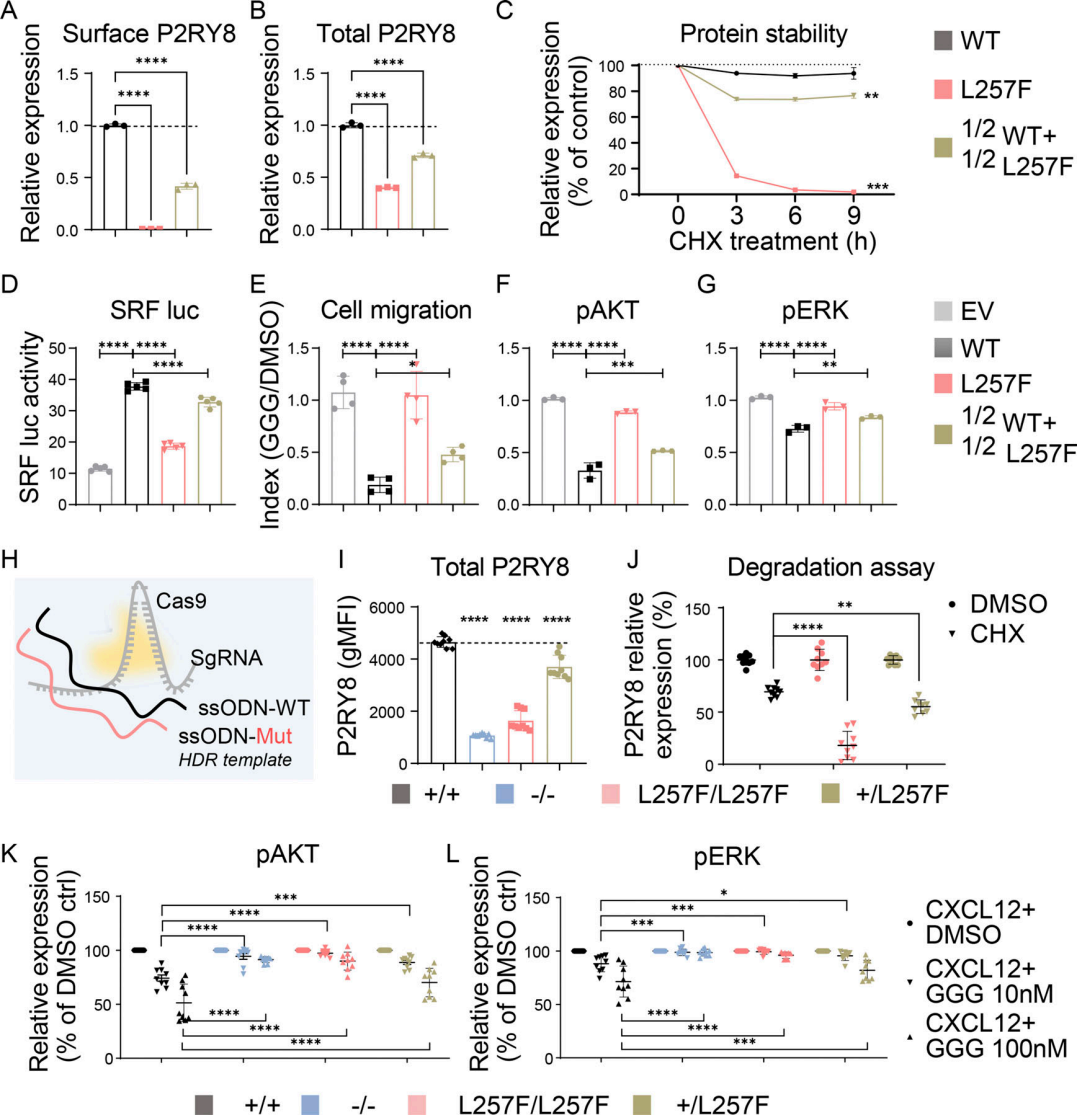
**De novo L257F variant causes P2RY8 haploinsufficiency. (A and B)** Flow cytometry analysis of P2RY8 expression in NIH/3T3 cells transduced with WT, L257F, or 50%WT+50%L257F. Dashed lines show the mean value of WT. **(A)** Surface staining of P2RY8 with anti-FLAG (*n* = 3 for each group). **(B)** Total P2RY8 staining with anti-P2RY8 (*n* = 3 for each group). **(C)** Relative P2RY8 expression after CHX treatment. Values are relative to time 0 and are means of three replicates. Dotted line shows the mean value of WT. **(D)** SRF luciferase (SRF luc) activity in HEK293 cells transfected with P2RY8-WT, P2RY8-L257F, or 50%WT+50%L257F vector (*n* = 5 for each group). **(E–G)** Purified splenic B cells were transduced with pMSCV-IRES-GFP II (pMIG II) retroviruses encoding P2RY8-WT, P2RY8-L257F, or 50%WT+50%L257F. Transduced cells were treated with CXCL12+DMSO or with CXCL12+GGG. Cell migration (E; *n* = 4 for each group), pAKT (F), or pERK (G) index (*n* = 3 for each group) was calculated as levels in CXCL12+GGG divided by levels in CXCL12+DMSO. **(H–L)** L257F variant was introduced into the Raji B cell line via CRISPR-Cas9–mediated homology directed repair (HDR). ssODN, single-stranded oligo donor; Mut, L257F mutant. **(I)** Flow cytometry analysis of P2RY8 expression in Raji CRISPR cell lines. Dashed line shows the mean value of +/+. **(J)** P2RY8 degradation assay following CHX treatment. **(K and L)** Summary graphs showing inhibitory effects of GGG in CXCL12-mediated AKT (K) and ERK (L) activation. Each group of H–L contains three biological samples, with three technical replicates per sample. Data representative of two (A–G, I, and J) or three (K and L) independent experiments. P values determined by one-way ANOVA with Dunnett's multiple comparisons test (A, B, D–F, and I) or by two-way ANOVA with Dunnett's multiple comparisons test (C and J–L). *, P < 0.05; **, P < 0.01; ***, P < 0.001; ****, P < 0.0001. Graphs depict mean with SD. ctrl, control; gMFI, geometric mean; sgRNA, single-guide RNA.

The *P2RY8* gene is located in the pseudoautosomal region of the X and Y chromosomes (Mullighan et al., 2009). This region escapes X-inactivation and is normally expressed in both sex chromosomes. To investigate whether heterozygosity for the P2RY8-L257F allele causes functional defects, we compared transfection of 100% P2RY8-WT, 100% P2RY8-L257F, or a 50%:50% mix of P2RY8-WT:P2RY8-L257F into NIH/3T3, HEK293, and splenic B cells. The 50%:50% mix mimics the heterozygous state of L257F mutation. We measured protein expression (Fig. 4, A and B), protein stability (Fig. 4 C), SRF luciferase activity (Fig. 4 D), GGG-mediated inhibition of cell migration (Fig. 4 E), and AKT/ERK activation (Fig. 4, F and G). The results for 50% P2RY8-L257F transfections were intermediate between 100% P2RY8-L257F and those of 100% P2RY8-WT. To investigate the effects of L257F heterozygosity when expressed from the endogenous locus, we used CRISPR/Cas9 editing to introduce the L257F variant into the human Raji B cell line (Fig. 4 H; and Fig. S2, D and E). Heterozygosity for the L257F variant decreased P2RY8 protein expression (Fig. 4 I and Fig. S2 F) and stability (Fig. 4 J), leading to reduced GGG-mediated inhibition of CXCL12-induced AKT activity (Fig. 4 K) and ERK activity (Fig. 4 L). Taken together, these results suggest that the de novo L257F variant causes P2RY8 haploinsufficiency.

### P2RY8 expression and function in primary lymphoid cells from probands carrying *P2RY8* variants

**Figure S3.**
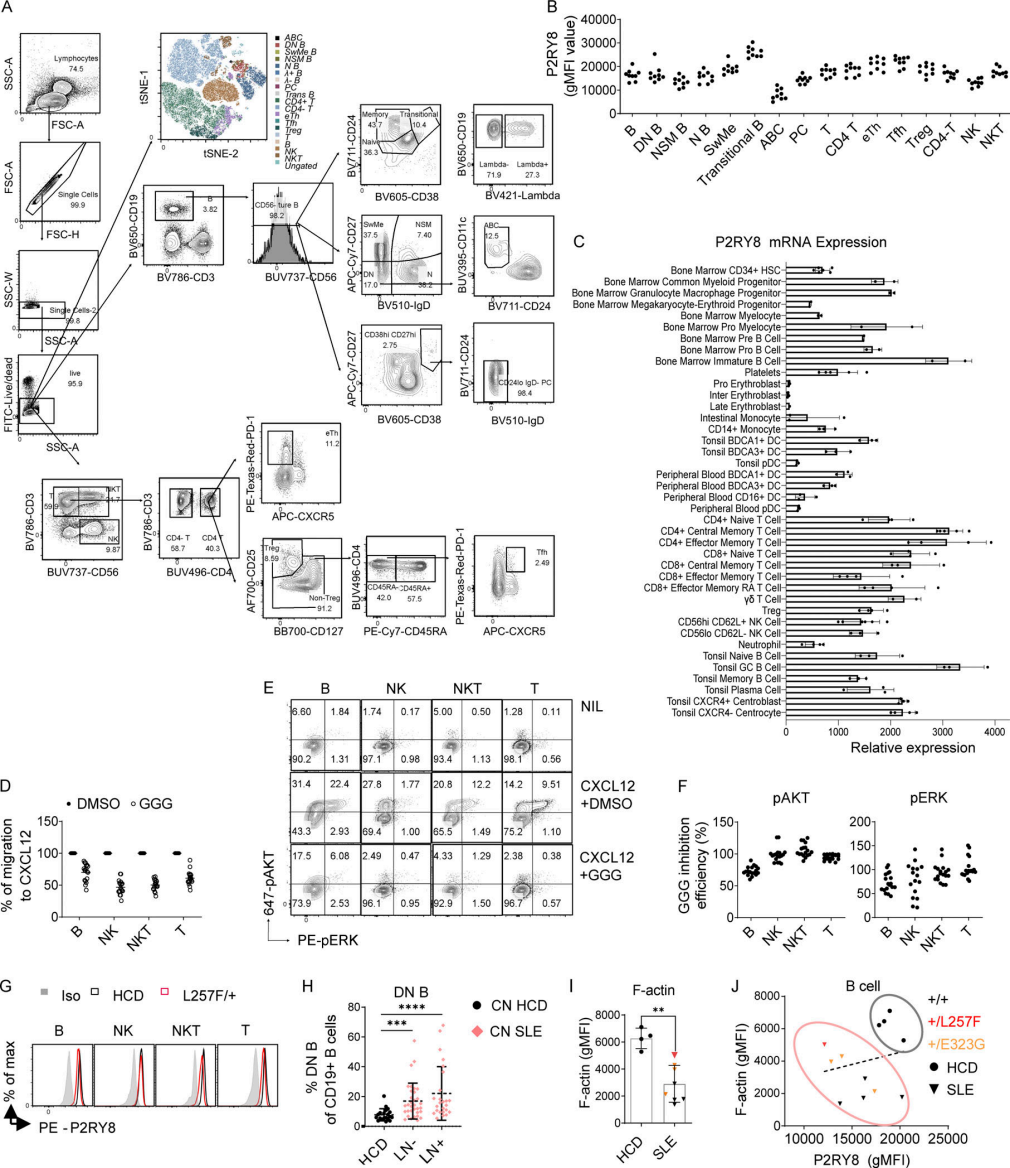
**GGG-mediated inhibition of AKT and ERK activation and cell migration in human PBMCs. (A)** Gating strategy for PBMC immunophenotyping. **(B)** Summary graph showing P2RY8 levels in different PBMC subsets. (*n* = 9 healthy subjects). **(C)** P2RY8 mRNA expression in immune cells. Expression data from BioGPS (Wu et al., 2013), using the Primary Cell Atlas database, a meta-analysis of publicly available microarray datasets derived from human primary cells. **(D)** Transwell migration assay toward 200 ng/ml CXCL12 along with 100 nM GGG (GGG) or vehicle (DMSO). **(E and F)** Representative FACS plots (E) and summary graphs (F) showing pAKT and pERK levels in B, natural killer (NK), natural killer T (NKT), or T cells treated with CXCL2+DMSO or CXCL12+GGG. (*n* = 9 subjects, two replicates for each subject.) GGG Inhibition Efficiency (%) = 100* ([CXCL12+GGG] %-NIL%)/([CXCL12+DMSO] %-NIL%). **(G)** Representative histograms showing P2RY8 expression in a healthy control and patient with +/L257F variant. Iso, isotype control. **(H)** Summary graph showing frequency of CD27 IgD DN B cells in CD19^+^ B cells. *n* = 20, HCD; *n* = 33, lupus nephritis (LN)^−^; *n* = 25, LN^+^. CN HCD, Chinese healthy donors; CN SLE, Chinese SLE patients. P values determined by Mann-Whitney test. **(I)** Summary graphs showing F-actin expression in B cells. *n* = 4, HCD; *n* = 8, SLE. Red dot, +/L257F (*n* = 1); orange dots, +/E323G (*n* = 3). **(J)** Correlation analysis between P2RY8 and F-actin expression levels in B cells. *n* = 4, HCD; *n* = 8, SLE. Red dot, +/L257F (*n* = 1); orange dots, +/E323G (*n* = 3). Data were representative of at least five (B–F) or four (H) independent experiments. P values determined by Mann-Whitney test (H and I) or by correlation analysis (J). **, P < 0.01; ***, P < 0.001; ****, P < 0.0001. Graphs depict mean with SD, and points represent biological replicates. ABC, age-associated B cells; eTh, extrafollicular helper T cells; effector memory RA T, effector memory T cells re-expressing CD45RA; FSC-A, forward scatter A; FSC-H, forward scatter H; gMFI, geometric mean; HSC, hematopoietic stem cells; inter, intermediate; max, maximum; N B, naive B cells; NIL, non-treated control; NK, Natural killer cells; NKT, Natural killer T cells; NSM B, non-switched memory B cells; PC, plasma cell; pro, progenitor; PE, phycoerythrin; SSC-A, side scatter A; SSC-W, side scatter W; SwMe, switched memory B cells; Trans B, transitional B cells; Treg, T regulatory cell; Tfh, T follicular helper cells.

Phenotyping of human peripheral blood mononuclear cells (PBMCs) showed that P2RY8 protein was widely expressed in lymphocytes including T cells, B cells, natural killer cells, and natural killer T cells (Fig. S3, A and B) in accord with public domain mRNA expression data (Fig. S3 C; Wu et al., 2013). Once engaged by its ligand GGG, P2RY8 functioned as an inhibitory receptor for each of these cell types when assessed for CXCL12-induced migration (Fig. S3 D) and AKT and ERK activation (Fig. S3, E and F). These observations broaden the potential sites of P2RY8 action well beyond GC B and T follicular helper cells.

**Figure 5.**
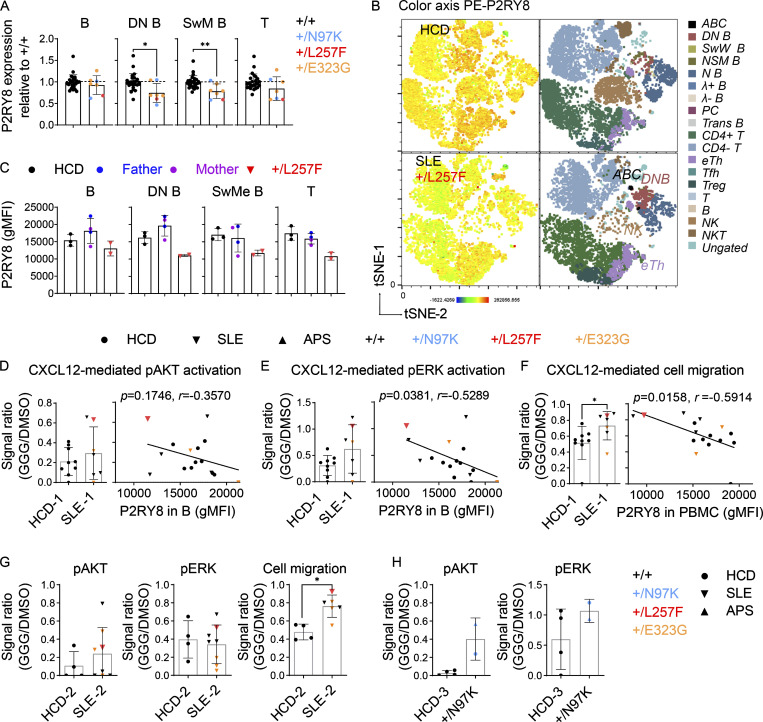
**PBMC immunophenotyping of patients with *P2RY8* variants. (A)** Summary graphs showing P2RY8 expression in B cells, DN B cells, switched memory (SwMe) B cells, and T cells relative to normal expression in +/+ (mean expression of +/+ was set as 1, dashed line). Black, +/+ (*n* = 25); blue, +/N97K (*n* = 2, APS proband and his mother); red, +/L257F (*n* = 1, SLE); orange, +/E323G (*n* = 4, SLE). **(B)** tSNE plots of PBMC clusters displaying P2RY8 expression in healthy control (HCD) versus patient with L257F variant. **(C)** Representative graphs showing P2RY8 expression in parents of +/L257F patient (*n* = 2 replicates for each donor, from different collection points), compared with that of age-matched healthy donors (*n* = 3 donors) and with that of SLE-diagnosed child (*n* = 2 replicates from different collection points). **(D and E)** Summary graph showing the inhibitory effect of GGG on CXCL12-induced pAKT (D) and pERK (E) expression in B cells from cohort 1 (*n* = 9, HCD-1; *n* = 7, SLE-1). Red, +/L257F; orange, +/E323G. Signal ratio was calculated as phosphorylation induced by CXCL12+GGG divided by mean value of phosphorylation induced by CXCL12+DMSO. Correlation analysis was performed between P2RY8 expression and signal ratio. **(F)** Transwell assay of PBMCs in cohort 1, migrated toward CXCL12+DMSO or CXCL12+GGG. Signal ratio was calculated as cell migration induced by CXCL12+GGG divided by cell migration induced by CXCL12+DMSO (*n* = 9, HCD-1; *n* = 7, SLE-1). Red, +/L257F; orange, +/E323G. Correlation analysis was performed between P2RY8 expression and migration signal ratio. **(G)** pAKT, pERK, or cell migration index in cohort 2. *n* = 4, HCD-2; *n* = 8, SLE-2 (pAKT and pERK); *n* = 6, SLE-2 (cell migration). Red, +/L257F; orange, +/E323G. **(H)** pAKT and pERK index in cohort 3. *n* = 4, HCD-3; *n* = 2, +/N97K. Data representative of five (A) independent experiments. P values determined by Mann-Whitney test (A, D left, E left, F left, G, and H), correlation analysis (D right, E right, and F right). *, P < 0.05; **, P < 0.01; ***, P < 0.001; ****, P < 0.0001. Graphs depict mean with SD, and points represent biological replicates. gMFI, geometric mean; ABC, age-associated B cells; N B, naive B cells; NSM B, non-switched memory B cells; PC, plasma cells; Trans B, transitional B cells; Treg, regulatory T cells; eTh, extrafollicular helper T cells; Tfh, T follicular helper cells; NK, natural killer cells; NKT, natural killer T cells.

The patient carrying the de novo L257F variant exhibited decreased P2RY8 expression across almost all lymphocyte subsets (Fig. 5, A and B; and Fig. S3 G), whereas PBMCs from her parents had P2RY8 expression comparable to that of healthy donors (Fig. 5 C). PBMCs from individuals heterozygous for N97K or E323G variants also had decreased P2RY8 expression in switched memory (SwM) B cells and B cells double-negative (DN) for CD27 and IgD that are typically expanded in lupus (Fig. S3 H and Fig. 5 A; Jenks et al., 2018; Wang et al., 2018).

Since F-actin is generally decreased in active SLE B cells (Fig. S3, I and J; Dong et al., 2015), downregulation of F-actin cannot be used as an indicator of P2RY8 defects in SLE B cells. We therefore focused on the other pathways we had characterized as being regulated by P2RY8 and GGG (Lu et al., 2019). Cells from the patient harboring the P2RY8-L257F variant showed a reduction in GGG-mediated inhibition of pAKT (Fig. 5 D) and pERK (Fig. 5 E) in B cells compared with healthy controls. This patient’s cells were also impaired in GGG-mediated inhibition of cell migration, resulting in higher cell migration to CXCL12 (Fig. 5 F). The GGG-dependent P2RY8 L257F–mediated functional defects were further confirmed in a second experiment with different SLE controls (Fig. 5 G). Moreover, low expression levels of P2RY8 correlated with high levels of chemokine-induced ERK activation (Fig. 5 E) and cell migration (Fig. 5 F) in the presence of GGG across all SLE patients.

PBMCs from the two donors harboring the N97K variant also showed decreased GGG responsiveness to CXCL12-mediated AKT and ERK activation (Fig. 5 H). Only two out of three patients harboring the less-rare E323G mutation showed decreased GGG-mediated inhibition of AKT activation and cell migration (Fig. 5, D–G; two patients were included in D–F and three in G). Collectively, these results suggest that heterozygosity for P2RY8 variants impairs protein function to different degrees, with the de novo and novel variants causing the most severe defects.

### P2RY8 is down-regulated in SLE

**Figure 6.**
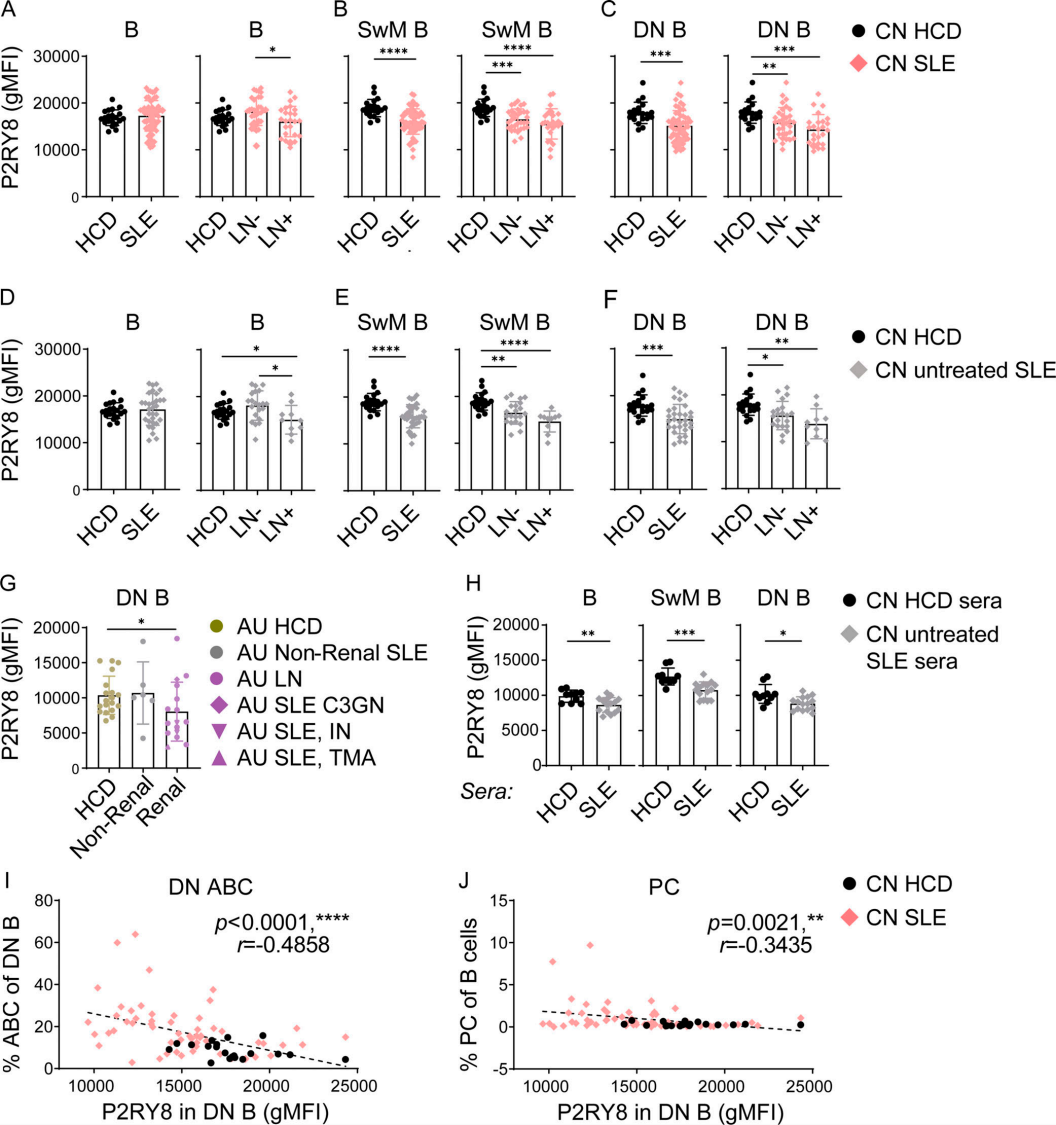
**Decreased P2RY8 expression in B cell subsets in lupus patients. (A–C)** Immunophenotyping of Chinese (CN) SLE patients. *n* = 20, HCD; *n* = 58, SLE; *n* = 33, lupus nephritis (LN)^−^; *n* = 25, LN^+^. Flow cytometry analysis of P2RY8 expression in B cells (A), SwM B cells (B), and DN B cells (C).** (D–F)** Immunophenotyping of untreated CN SLE patients. *n* = 20, HCD; *n* = 31, SLE; *n* = 21, LN^−^; *n* = 10, LN^+^. Flow cytometry analysis of P2RY8 expression in B cells (D), SwM B cells (E), and DN B cells (F).** (G)** Immunophenotyping of AU non-Chinese SLE patients (*n* = 19, HCD; *n* = 6, non-renal SLE; *n* = 16, renal SLE). C3GN, C3 glomerulonephritis; IN, interstitial nephritis; TMA, thrombotic microangiopathy. Summary graphs showing P2RY8 expression in DN B cells. **(H)** 2 × 10^5^ purified HCD B cells were cultured in 150 µl RPMI 1640 medium containing 10% serum from HCD or untreated SLE patients. P2RY8 expression was analyzed 72 h after stimulation. *n* = 10, HCD sera; *n* = 15 SLE sera. **(I and J)** Correlation analysis between DN ABC (I) or PC frequency (J) and P2RY8 levels in DN B cells. *n* = 20, CN HCD; *n* = 58, CN SLE. Data representative of four (A–C, I, and J) or two (D–F and H) independent experiments. P values determined by Mann-Whitney test (A–H) or by correlation analysis (I and J). *, P < 0.05; **, P < 0.01; ***, P < 0.001; ****, P < 0.0001. Graphs depict mean with SD, and points represent biological replicates. gMFI, geometric mean; PC, plasma cell.

**Figure S4.**
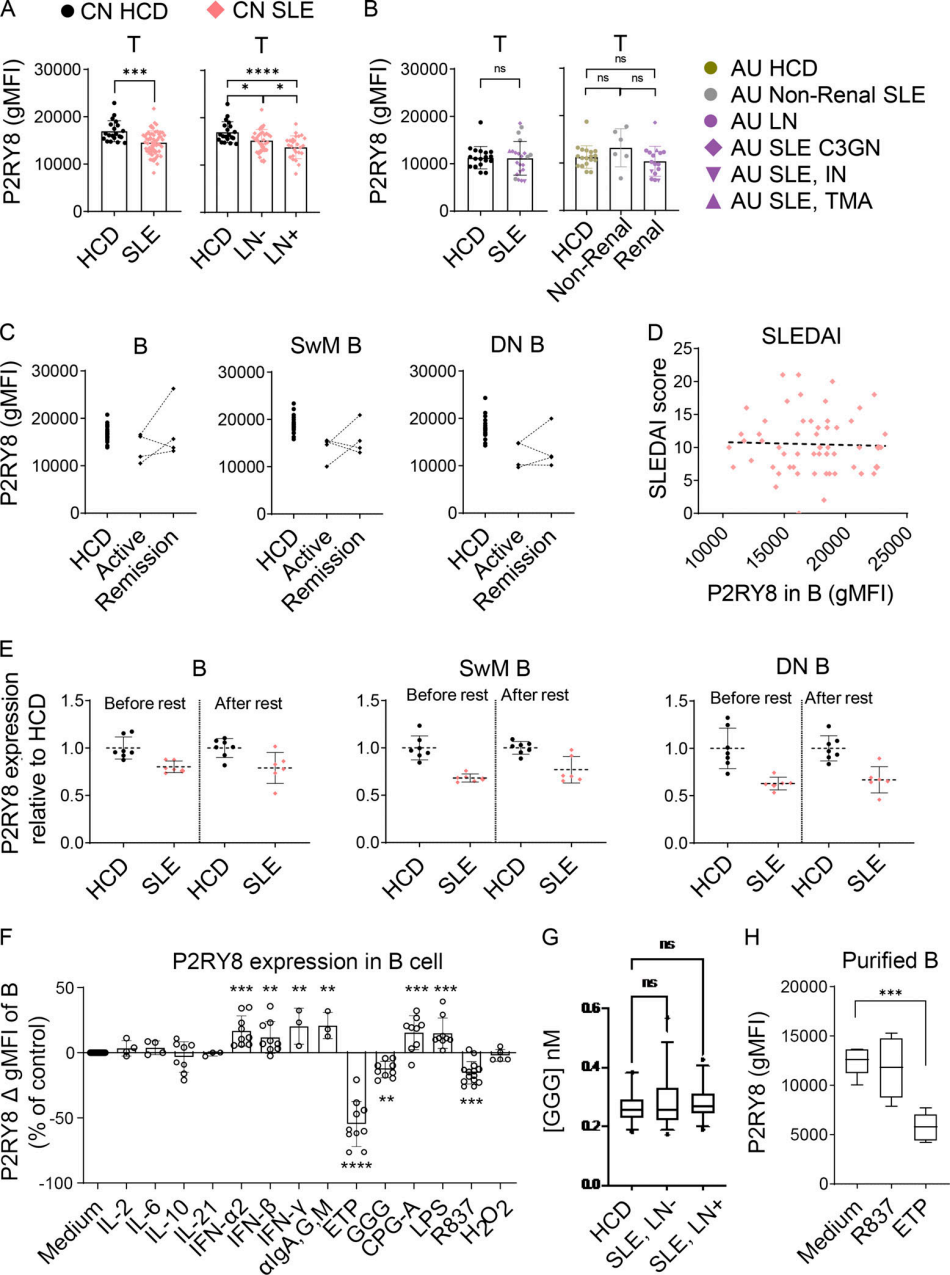
**P2RY8 is down-regulated in B cell subsets of SLE patients. (A)** Flow cytometry analysis of P2RY8 expression in T cells of Chinese (CN) SLE patients. *n* = 20, HCD; *n* = 54, SLE; *n* = 30, lupus nephritis (LN)^−^; *n* = 24, LN^+^. **(B)** Immunophenotyping of AU non-Chinese SLE patients (*n* = 19, HCD; *n* = 6, non-renal SLE; *n* = 16, renal SLE). C3GN, C3 glomerulonephritis; IN, interstitial nephritis; TMA, thrombotic microangiopathy. Summary graphs showing P2RY8 expression in T cells. **(C)** Summary graphs showing P2RY8 expression in B cells, SwM B, and DN B cells in SLE patients during active disease course (active) or after remission induction (remission). *n* = 20, HCD; *n* = 4, SLE (active and remission). **(D)** Correlation analysis between SLEDAI score and P2RY8 expression levels of B cells in CN SLE patients (*n* = 58). **(E)** PBMCs were washed twice and rested in B cell culture medium for 24 h. Summary graphs showing P2RY8 expression in B cells, SwM B, and DN B cells before and after rest, compared with normal expression in HCD (mean expression of HCD was set as 1). *n* = 7, HCD; *n* = 6, SLE. **(F)** 3 × 10^5^ PBMCs in 200 µl cell culture medium were untreated (Medium, *n* = 14) or treated as indicated with IL-2 (100 U/ml, *n* = 4), IL-6 (20 ng/ml, *n* = 4), IFN-α2 (50 ng/ml, *n* = 9), IFN-β (50 ng/ml, *n* = 9), LPS (1 µg/ml, *n* = 9), or H_2_O_2_ (100 µM, *n* = 5) for 24 h or with IL-10 (50 ng/ml, *n* = 8), IL-21 (50 ng/ml–, *n* = 3), IFN-γ (50 ng/ml, *n* = 3), αIgA, G, M (5 µg/ml, *n* = 3), ETP (50 µM, *n* = 7), GGG (100 nM, *n* = 9), R837 (5 µg/ml, *n* = 14), or CPG-A (0.5 µM, *n* = 9) for 48 h. Summary graphs showing the expression of P2RY8 in live B cells after stimulation. **(G)** Summary graphs showing GGG concentration in serum from HCD versus SLE patients. *n* = 30, HCD; *n* = 30, SLE without lupus nephritis (SLE, LN^−^); *n* = 30, SLE with lupus nephritis (SLE, LN^+^). **(H)** 2 × 10^5^ purified B cells in 200 µl cell culture medium were untreated (Medium, *n* = 6) or treated with R837 (*n* = 6) or ETP (*n* = 6) for 72 h. Summary graphs showing the expression of P2RY8 in live B cells after stimulation. Data representative of four (A and D), two (E and H), or at least five (F) independent experiments. P values determined by Mann-Whitney test (A and B), correlation analysis (D), or ANOVA (F–H). *, P < 0.05; **, P < 0.01; ***, P < 0.001; ****, P < 0.0001. Graphs depict mean with SD, and points represent biological replicates. gMFI, geometric mean.

We identified some SLE patients with very low P2RY8 expression in B cells, despite lacking mutations in the coding region of *P2RY8*. Downregulation of P2RY8 was more prominent in patients with lupus nephritis (Fig. 6 A). Remarkably, SLE patients as a group had decreased P2RY8 expression in SwM B cells (Fig. 6 B) and DN B cells (Fig. 6 C), as well as in T cells (Fig. S4 A). To exclude the influence of medication on the observed phenotypes, we analyzed PBMCs from 31 untreated SLE patients. In this group, downregulation of P2RY8 in B cells (Fig. 6 D), SwM B cells (Fig. 6 E), and DN B cells (Fig. 6 F) was also observed. Moreover, a similar downregulation of P2RY8 in DN B cells (but not in T cells) was seen in AU non-Asian SLE patients with renal involvement (Fig. 6 G and Fig. S4 B), suggesting that P2RY8 downregulation can be extrapolated to non-Chinese SLE and appears to serve as a biomarker of renal lupus. Together, these data raised the possibility that P2RY8 downregulation may be involved more broadly in SLE pathogenesis.

To investigate whether P2RY8 downregulation occurs predominantly during periods of disease activity, we compared the expression of P2RY8 in four follow-up patients bled during periods of disease remission and relapse. Two out of four SLE patients showed increased P2RY8 expression in SwM and DN B cells during remission, although only one of them reached normal expression (Fig. S4 C). There was no correlation between P2RY8 expression and Systemic Lupus Erythematosus Disease Index (SLEDAI) score (Fig. S4 D), suggesting P2RY8 downregulation is a fixed phenotype in SLE. P2RY8 expression did not increase either after resting the cells, further suggesting downregulation of P2RY8 is not a transient process but a relatively stable phenotype (Fig. S4 E).

We considered the possibility that downregulation of P2RY8 may occur in part due to signals from circulating factors. Consistent with this, treatment of purified B cells with patients’ serum lowered P2RY8 expression (Fig. 6 H). GGG has been shown to cause a decrease in P2RY8 by driving endocytic degradation (Fig. S4 F; Lu et al., 2019), but we could not detect differences in serum GGG concentration between healthy control donors (HCDs) and SLE (Fig. S4 G). Our in vitro studies did not support that reduced P2RY8 expression in SLE patients was a consequence of elevated IFN or BCR signaling (Fig. S4 F). A trial of multiple B cell stimuli, including various cytokines and TLR ligands, revealed that etoposide (ETP), an inducer of DNA damage and apoptosis, consistently decreased P2RY8 abundance in cultured B cells (Fig. S4, F and H). The TLR7 agonist R837 could also modestly down-regulate P2RY8 in B cells (Fig. S4 F). Similar downregulation could not be observed when stimulating purified B cells (Fig. S4 H), suggesting an indirect effect of TLR7 on B cells, possibly via acting on myeloid cells. Downregulation of P2RY8 in SLE is likely to be a result of a combination of multiple factors including DNA damage and exposure to TLR ligands.

### Low P2RY8 correlates with nephritis, DN age-associated B cells (ABCs), and plasma cells

To further explore the possible links between P2RY8 reduction and disease pathogenesis, SLE patients were divided into those with high or low expression of P2RY8 in B cells compared with normal expression in healthy donors, and clinical and immunological features were then compared between those two groups. SLE patients with low P2RY8 expression had a statistically significant increase in the incidence of nephritis (Table 1), though disease activity indicated by SLEDAI scores was not different (Table 1).

**Table 1.** Clinical features of P2RY8^low^ and P2RY8^hi^ patients

**Features**	**SLE patients without P2RY8 variants**			**Patients with P2RY8 variants**		
	**P2RY8^low^** **(*n* = 22)**	**P2RY8^hi^** **(*n* = 31)**	**P value**	**+/L257** **(*n* = 1)**	**+/N97K** **(*n* = 1)**	**+/E323G** **(*n* = 6)**
Diagnosis	SLE	SLE		SLE	APS	SLE
Sex, female	100% (22/22)	100% (31/31)	N/A	100% (1/1)	0% (0/1)	83% (5/6)
Age at onset (yr)	24.41 ± 9.12	23.80 ± 9.82	0.8754	13	45	28.83 ± 18.99
SLEDAI	10.82 ± 5.41	10.31 ± 4.12	0.7354	12	N/A	7.67 ± 3.50
Alopecia	32% (7/22)	29% (9/31)	0.7703	0% (0/1)	0% (0/1)	17% (1/6)
Arthritis	64% (14/22)	45% (14/31)	0.1843	100% (1/1)	0% (0/1)	50% (3/6)
Serositis	5% (1/22)	0% (0/31)	0.2308	0% (0/1)	0% (0/1)	17% (1/6)
Leukopenia	23% (5/22)	39% (12/31)	0.2193	0% (0/1)	0% (0/1)	17% (1/6)
Lymphopenia	50% (11/22)	33% (10/30)	0.2262	0% (0/1)	100% (1/1)	17% (1/6)
Renal	64% (14/22)	32% (10/31)	**0.0237**	100% (1/1)	0% (0/1)	33% (2/6)
ANA	100% (21/21)	100% (28/28)	N/A	100% (1/1)	100% (1/1)	100% (6/6)
Anti-dsDNA	68% (15/22)	45% (14/31)	0.0971	100% (1/1)	0% (0/1)	83% (5/6)
Anti-SM	32% (7/22)	13% (4/30)	0.1069	100% (1/1)	0% (0/1)	33% (2/6)
Anti-RNP	40% (8/20)	26% (7/27)	0.3061	100% (1/1)	0% (0/1)	33% (2/6)
Anti-SSA	50% (11/22)	52% (16/31)	0.9079	100% (1/1)	0% (0/1)	50% (3/6)
Anti-SSB	14% (3/22)	13% (4/31)	0.9381	0% (0/1)	0% (0/1)	17% (1/6)
Cardiolipin antibodies	0% (0/22)	3% (1/29)	0.3790	0% (0/1)	100% (1/1)	17% (1/6)
β-2 glycoprotein I antibodies	8% (1/12)	16% (3/19)	0.5464	0% (0/1)	100% (1/1)	25% (1/4)
Consumed C3	86% (18/21)	90% (27/30)	0.6401	100% (1/1)	0% (0/1)	83% (5/6)
Consumed C4	81% (17/21)	63% (19/30)	0.1741	100% (1/1)	0% (0/1)	83% (5/6)
**Medication**				**MP, HCQ, MMF**	**Warfarin**	
MP	14% (3/21)	27% (8/30)	0.2901	100% (1/1)	0% (0/1)	33% (2/6)
PDN	29% (6/21)	27% (8/30)	0.8808	0% (0/1)	0% (0/1)	67% (4/6)
HCQ	19% (4/21)	17% (5/30)	0.8263	100% (1/1)	0% (0/1)	33% (2/6)
MMF	9.5% (2/21)	13% (4/30)	0.6777	100% (1/1)	0% (0/1)	33% (2/6)

P2RY8^low^, geometric mean (gFMI) in B cells < 16,864 (mean value of P2RY8 gMFI in B cells from healthy control donors); P2RY8^hi^, P2RY8 gMFI in B cells > 16,864. Data were presented in the form of mean ± SD or % (*n*/total). P value was determined by Mann-Whitney test or by χ^2^ test. A P value <0.05 is considered statistically significant and is bolded. C3, complement C3; C4, complement C4; MP, methylprednisolone; N/A, not applicable; PDN, prednisone; RNP, ribonucleoprotein; SS, Sjogren syndrome.

A subset of DN B cells also express CD11c and are known as ABCs or DN2 cells. ABCs are considered to be pathogenic in lupus nephritis (Jenks et al., 2018; You et al., 2020) and have been suggested to represent a major source of autoantibody-secreting cells (Jenks et al., 2018; Wang et al., 2018). ABCs were largely expanded in SLE, consistent with other reports (Jenks et al., 2018; You et al., 2020), and the expansion was more prominent in SLE patients with low expression of P2RY8 such that there was a negative correlation between P2RY8 and ABC frequency (Fig. 6 I).

Increased circulating plasmablasts can sometimes be seen in SLE. We found a modest inverse correlation between P2RY8 expression and the frequency of circulating CD38^hi^CD27^hi^CD24^lo^IgD^−^ plasmablasts (Fig. 6 J). P2RY8^low^ patients also exhibited higher anti-dsDNA and anti-SM autoantibodies, though this did not reach statistical significance (Table 1). Together, these data provide an intriguing link between P2RY8, antibody responses, and end-organ (renal) damage.

### P2RY8 can restrain plasma cell formation

**Figure 7.**
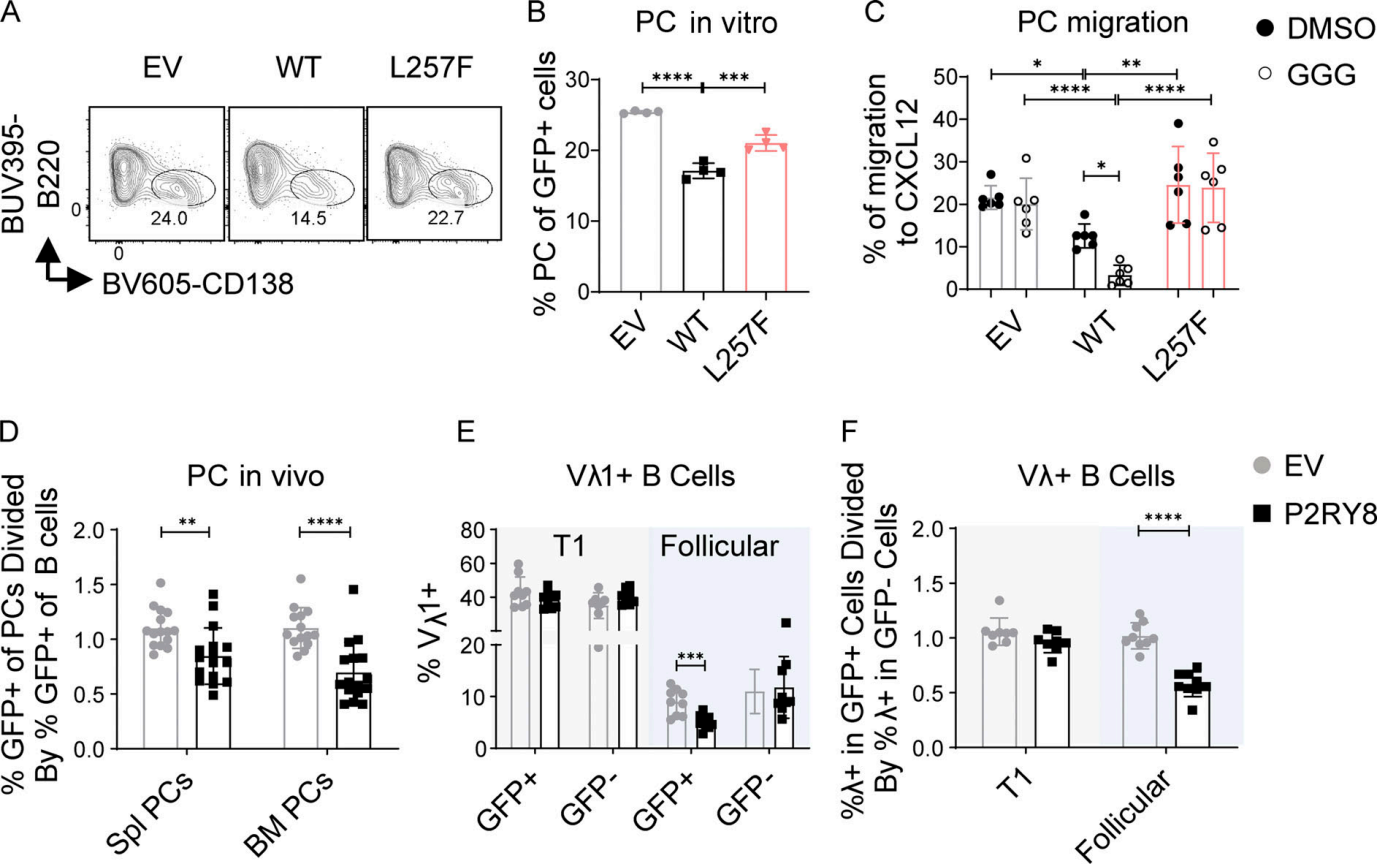
**P2RY8 restrains plasma cell formation and enforces B cell–negative selection. (A–C)** Purified splenic B cells were preactivated by LPS and transduced with retroviruses encoding P2RY8-IRES-GFP or the L257F mutant. **(A and B)** Representative plots (A) and summary graph (B) showing the frequency of CD138^+^ plasma cells (PCs) in indicated retrovirally transduced B cells (*n* = 4 for each group). **(C)** Transwell assay of CD138^+^ plasma cells toward CXCL12 (DMSO, *n* = 6) or with CXCL12+GGG (GGG, *n* = 6). **(D–F)** Bone marrow (BM) from WT (D) or V_H_3H9-Tg mice (E and F) retrovirally transduced with EV-IRES-GFP or P2RY8-IRES-GFP was used to reconstitute congenically distinct mice. **(D)** The mice were immunized 8–12 wk after reconstitution, on day 0 and day 7 with SRBC i.p., and then the tissue was harvested on day 10. GFP^+^ cell representation was determined by flow cytometry and plotted as a ratio of GFP^+^ frequency in plasma cells divided by GFP^+^ frequency in splenic (Spl) B cells for each independent mouse (EV, *n* = 15; P2RY8, *n* = 16). **(E and F)** Tissues were harvested at 8–12 wk following reconstitution. **(E)** V_λ_1 usage in splenic developmental B cell subsets determined in GFP^+^ and GFP^−^ cells. **(F)** Summary graph showing ratio of λ usage between GFP^+^ and GFP^−^ populations. GFP^−^ (untransduced) cells serve as an internal control (*n* = 9, except for T1:GFP^−^, where *n* = 8). Data representative of two (C), three (D–F), or at least five (A and B) independent experiments. P values determined by one-way ANOVA with Dunnett's multiple comparisons test (B) or two-way ANOVA with Sidak's multiple comparisons test (C) or by unpaired two-tailed Student’s test (D–F). *, P < 0.05; **, P < 0.01; ***, P < 0.001; ****, P < 0.0001. Graphs depict mean with SD. EV, empty vector.

**Figure S5.**
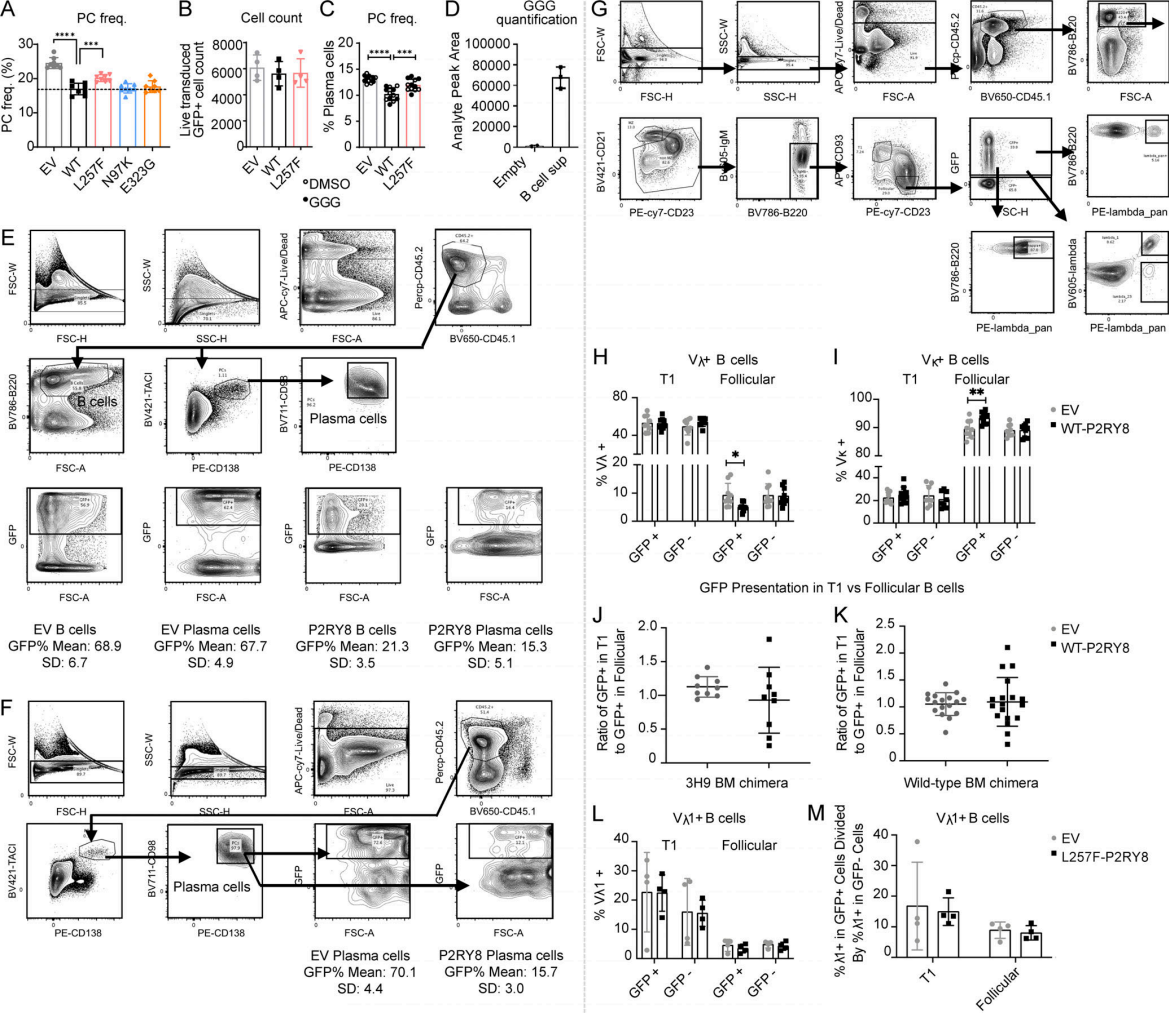
**P2RY8 restrains plasma cell formation and enforces B cell–negative selection. (A–D)** Purified splenic B cells were activated with LPS for 24 h and then transduced with retroviruses encoding WT-P2RY8 or mutants. **(A)** Summary graph showing the frequency of CD138^+^ plasma cells in indicated retrovirally transduced B cells (*n* = 8 for each group). **(B)** Summary graph showing the total number of live GFP^+^ cells 2 d after transduction (*n* = 4 for each group). **(C)** Summary graph showing the frequency of CD138^+^ plasma cells in B cells retrovirally transduced with WT-P2RY8–WT-IRES-Thy1.1 or mutants. Cultures were treated with vehicle (DMSO, open circles) or GGG (closed circles). **(D)** Purified splenic B cells were activated with LPS, and GGG levels were measured in the culture supernatant 96 h following activation using LC-MS/MS. Empty refers to LPS-containing media incubated in neighboring wells with no B cells (*n* = 3). **(E and F)** Gating strategy and chimerism for plasma cell analysis of spleen and bone marrow (BM) of P2RY8-GFP chimeras. Bone marrow retrovirally transduced with empty vector–IRES-GFP (EV) or WT P2RY8-IRES-GFP was used to reconstitute congenically distinct mice. Spleens (E) and bone marrow (F) were harvested from these mice at 8–12 wk following reconstitution, and GFP^+^ cell frequencies in B cells and plasma cells were determined by flow cytometric gating. Mean and SD for GFP frequency listed beneath EV and P2RY8 plots. *n* = 6, with data representative of three independent experiments. **(G**–**M)** V_H_3H9 Tg mouse BM retrovirally transduced with EV-GFP, WT-P2RY8-GFP, or L257F-P2RY8-GFP was used to reconstitute congenically distinct mice. Spleens were harvested from these mice at 9–12 wk following reconstitution, with splenic developmental B cell subsets and V_λ_, V_λ_1, and V_κ_ usage determined by flow cytometry. **(G)** FACS profiles show gating strategy for V_λ_, V_λ_1, and V_κ_ analysis of V_H_3H9 Tg mice. **(H and I)** Summary graphs of WT-P2RY8-GFP and EV-GFP experimental animals showing frequency of V_λ_ (H) and V_κ_ (I) cells within the indicated subsets (*n* = 9 except for T1:GFP^−^ where *n* = 8). **(J and K)** Summary graph showing ratio of total GFP^+^ cells in the T1 compartment relative to the follicular compartment in the 3H9 BM chimeras (J) and WT BM chimeras (K).** (L)** Summary graph showing Vλ1 usage in L257F-P2RY8-GFP and EV-GFP animals. **(M)** Summary graph showing ratio of Vλ usage between GFP^+^ and GFP^−^ populations in L257F-P2RY8-GFP and EV-GFP animals. GFP^−^ (untransduced) cells serve as an internal control (*n* = 4). Data were representative of three (E–M) or at least five independent experiments (A–C). P values determined by one-way ANOVA (A and C) or by unpaired two-tailed Student’s test (H–M). *, P < 0.05; **, P < 0.01; ***, P < 0.001; ****, P < 0.0001. Graphs depict mean with SD. freq., frequency; FSC-A, forward scatter-A; FSC-H, forward scatter-H; FSC-W, forward scatter-W; PC, plasma cell; SSC-H, side scatter-H; SSC-W, side scatter-W.

Active PI3K/AKT and ERK are known to promote B cell differentiation into antibody-secreting cells or plasma cells (Lau et al., 2020; Omori et al., 2006; Yasuda et al., 2011). Since P2RY8 inhibited AKT and ERK signaling in the presence of GGG and plasmablasts were higher in patients with low P2RY8 expression in B cells, we speculated that P2RY8 itself might play a role in limiting plasma cell development. To test this, LPS-preactivated mouse B cells were retrovirally transduced with WT or mutant P2RY8 or with empty vector as a control. P2RY8 expression decreased the frequency of plasma cells in culture (Fig. 7, A and B; and Fig. S5 A) relative to total cell numbers (Fig. S5 B). Similar results were obtained using a different retroviral vector (IRES-Thy1.1 murine stem cell virus [MSCV]; Fig. S5 C). The L257F variant was less effective at inhibiting plasma cell differentiation in LPS-stimulated B cells (Fig. 7, A and B); the other variants did not show an effect in this assay (Fig. S5 A). P2RY8 L257F also failed to support GGG-mediated inhibition of plasma cell migration toward CXCL12 (Fig. 7 C), which may favor plasma cell infiltration into inflamed tissues such as kidneys (Cheng et al., 2018; Wang et al., 2009). Interestingly, administration of GGG did not further repress plasma cell differentiation (Fig. S5 C). Given that cultured B cells themselves were able to produce GGG (Fig. S5 D), autocrine or paracrine GGG is probably sufficient to engage P2RY8 and inhibit plasma cell differentiation.

To examine the influence of P2RY8 in plasma cell formation in vivo, bone marrow chimeric mice were generated that express P2RY8 in approximately one fifth of their B cells (Fig. S5, E and F). Following immunization, cells expressing P2RY8 were underrepresented in the spleen and bone marrow plasma cell compartments (Fig. 7 D). These data indicate that P2RY8 has the capacity to restrain signals, leading to plasma cell formation or accumulation.

### P2RY8 enforces B cell–negative selection in a mouse model

Elevated PI3K/AKT signaling promotes autoimmunity (Barber et al., 2005; Ge et al., 2017; Stylianou et al., 2011; Wu et al., 2014). This is driven, at least in part, by a B cell–intrinsic tolerance defect that impairs counterselection of self-reactive transitional B cells into the recirculating mature B cell pool (Greaves et al., 2019). To explore the hypothesis that P2RY8 expression promotes tolerance in developing B cells, we examined the effect of expressing P2RY8 in mice that also express the DNA-reactive V_H_3H9 heavy chain that is derived from a lupus-prone mouse strain (Chen et al., 1995). V_H_3H9 expression results in a bias of the B cell repertoire toward DNA reactivity when paired with endogenous λ light chains, particularly upon pairing with λ-1 (Fields et al., 2005). Mice were reconstituted with V_H_3H9 transgenic bone marrow that had been transduced with P2RY8-GFP or control-GFP retroviral vectors. Notably, WT P2RY8 expression reinforced negative selection at the T1 to follicular B cell transition, leading to decreased frequencies of λ-1^+^ and total λ^+^ follicular B cells (Fig. 7, E and F; and Fig. S5, G–I). P2RY8 did not alter the overall T1 to follicular transition (Fig. S5, J and K). The tolerance-promoting effect of P2RY8 was abrogated upon expression of the L257F variant (Fig. S5, L and M).

## Discussion

Our study has characterized an allelic series of rare variants of *P2RY8* and uncovered a new role of this receptor in immune tolerance, likely to be relevant for human systemic autoimmune diseases. Our data and earlier findings (Mullighan et al., 2009; Palmi et al., 2012; Vesely et al., 2017) indicate that P2RY8 is well expressed by immature B cells. Evidence that increased PI3K/AKT activity perturbs negative selection of immature B cells into the recirculating pool (Greaves et al., 2019), together with our data showing P2RY8 reduces accumulation of DNA-reactive follicular B cells in a mouse model, suggests P2RY8 may be important for this tolerance checkpoint. Alterations in lymphocyte migration and the actin cytoskeleton have also been implicated in systemic autoimmune disease development (Bouma et al., 2009; Chong and Mohan, 2009; Cook et al., 2020), and deregulated migration of P2RY8 mutant B cells at various stages of the antibody response may also contribute to pathogenesis. Furthermore, ERK signaling is important for plasma cell generation (Yasuda et al., 2011), and the increased ability of this pathway to be activated when P2RY8 is reduced may contribute to a less-stringent tolerance checkpoint during plasma cell formation.

Our finding that many SLE patients show reduced P2RY8 expression is striking, but it also hampers our ability to show a selective influence of the coding variants on endogenous P2RY8 function in PBMCs. However, since the receptor variants had diminished function in multiple systems, we believe that the most parsimonious explanation of our findings is that P2RY8 function can be compromised in lupus and APS patients by mutations in the coding region or by certain environmental factors, leading to P2RY8 reduction. Our data suggest that conditions leading to DNA damage as well as TLR7 receptor ligands may contribute to P2RY8 reduction.

To date, the function of P2RY8 in B cells has been mainly studied in the context of GCs (Muppidi et al., 2015; Muppidi et al., 2014). Although somatic hypermutation can contribute to both gain and loss of self-reactivity and autoimmunity may be caused by both follicular and extrafollicular pathways, molecular defects (i.e., DNASE1L3 and IKZF1 deficiency) have been shown to drive extrafollicular SLE (Al-Mayouf et al., 2011; Jenks et al., 2019; Schwickert et al., 2019; Soni et al., 2020; Van Nieuwenhove et al., 2018). Elevated CXCR4 expression has been previously observed in lupus patients’ B cells (Chong and Mohan, 2009; Hanaoka et al., 2015; Zhao et al., 2017). Despite its important role in organizing GC dark and light zones, the CXCR4 ligand CXCL12 is more abundantly expressed in extrafollicular regions than within GCs (Allen et al., 2004). A possible consequence of P2RY8/CXCR4 disturbance (whether caused by reduced P2RY8 or increased CXCR4 or both) is to prevent GC confinement of pathogenic B cells and thus avoid the various GC tolerance checkpoints, while favoring B cell localization to extrafollicular foci (Biajoux et al., 2016; Hargreaves et al., 2001). Moreover, decreased inhibition of CXCR4/CXCL12 signaling by P2RY8 may synergize with higher CXCR4 expression in deregulating AKT and ERK activity. CXCR4 also appears crucial for pathogenic B cell accumulation in the kidneys in lupus nephritis (Wang et al., 2009). Thus, P2RY8 signaling may function as a tolerance checkpoint that acts via GC retention and by restriction of plasma cell trafficking. The basis for the negative correlation between P2RY8 expression and DN B cell or ABC frequency is currently less clear but might also be a consequence of increased extrafollicular responses or altered signaling.

The proband with the P2RY8-L257 variant did not carry other potentially damaging de novo variants or rare variants in genes known to cause monogenic autoimmunity; only a low-frequency heterozygous *DNASE1* variant, Q31E, was identified, inherited from her father. This variant is found in ∼1:100 EASs (EAS ExAC MAF = 0.0052); we found this variant in two unrelated healthy Chinese controls out of 166 sequenced. The father was healthy and did not present any of the phenotypic abnormalities found in SLE. Together, this suggests that this *DNASE1* variant is highly unlikely to cause the severe childhood-onset SLE, but it is possible that it contributes to disease.

P2RY8 variants are likely to exert a spectrum of functional effects, with the de novo L257F variant being the strongest one and conferring complete loss of function, ultra-rare N97K being intermediate in its effects, and the less-rare E323G being much milder. Each of these variants is likely to act in the context of other genetic or environmental risk factors, with the E323G variant having the greatest dependence on additional factors.

Collectively, our study suggests P2RY8 has roles in multiple immune tolerance checkpoints, including promoting GC confinement, restraining plasma cell accumulation, and limiting selection of immature self-reactive B cells into the recirculating pool. Future studies in humanized mouse models may help further delineate the sites of P2RY8 action in preventing systemic autoimmune disease. Finally, our findings raise the possibility that augmenting signaling via the P2RY8 pathway may have therapeutic potential in prevention or treatment of systemic autoimmune disease.

## Materials and methods

### Human samples and sequencing

All human samples from Chinese, AU, and US patients were obtained under ethics committee approvals from Renji Hospital Shanghai Jiaotong University School of Medicine, Australian National University, and University of California, San Francisco. All donors signed informed consents. Genomic DNA was isolated from blood using QIAamp DNA Blood kit (QIAGEN) and was sequenced by Illumina platform. Bioinformatic analyses were performed at China-Australia Centre for Personalised Immunology. Relatedness was checked using Peddy (Pedersen and Quinlan, 2017). Identified variants were scored based upon a predesigned algorithm incorporating frequency of single-nucleotide polymorphism (MAF < 0.005), genetic tolerance of resulting amino acid changes (dn/ds metric), in silico predictions of damage (PolyPhen, SIFT, and CADD [score > 12]), and function of domains bearing mutation. Variants with low minor allele count (<5) were filtered out. All variants of interest in P2RY8 were further confirmed by Sanger sequencing. Detailed patient characteristics are presented in Table 1 and Table S1.

### Expression vectors and mutagenesis

GFP-tagged P2RY8 vector was obtained from Sino Biological (Cat #HG22967-ACGLN) or made as previously described (Muppidi et al., 2014). P2RY8 retroviral constructs were made by inserting Flag-tagged cDNA into retroviral vectors, including IRES-GFP containing PMIGII vector and IRES-Thy1.1 containing MSCV2.2 vector. Mutant vectors were constructed according to QuickChange site-directed mutagenesis protocols (Agilent Technologies).

### Retroviral transduction

Retrovirus was packaged using PLAT-E cells and was concentrated to 20-fold with PEG Virus Precipitation Kit (Yeasen; Cat #41101ES50). For NIH/3T3 cell transduction, cells were placed in 24-well plates and cultured overnight with retrovirus medium consisting of 1/10 volume of retrovirus stock and polybrene at a final concentration of 8 µg/ml. For transduction of B cells, freshly isolated splenic B cells were purified using MojoSort Mouse Pan B Cell Isolation Kit (BioLegend; Cat #480052) or EasySep Streptavidin-conjugated Rapidspheres (EasySep; Cat #50001) by removing T cells with biotin-conjugated anti-CD3e (BioLegend; clone 145-2C11). Purified B cells were cultured in 6-well plates and activated by 10 µg/ml LPS or 0.25 µg/ml anti-CD180 (BD Biosciences; 552128), diluted in RPMI 1640 containing 10% FBS, 10 mM Hepes, 55 µM β-mercaptoethanol, 2 mM glutamine, and 50 IU penicillin/streptomycin. 24 h after activation, the plate was centrifuged, and the culture supernatant was saved. Hepes and polybrene were added to the retrovirus to a concentration of 20 mM and 2 µg/ml, respectively. This retroviral medium was used to spinfect the cells at 2,500 rpm for 2 h at room temperature; the viral supernatant was aspirated, and the original culture supernatant was returned to the cells. This spinfection was repeated for a second time 24 h later. 24 h after the second spinfection, cells were collected from each plate and washed twice, with transduction efficiency checked via flow cytometry.

### CRISPR/Cas9 editing of Raji human B cell line

Single-guide RNA construct was prepared by cloning the oligo pairs encoding the 20-nt guide sequences into the PX458 plasmid (pSpCas9 [BB]-2A-GFP; Addgene). WT and mutant homology directed repair templates that both possess a silent mutation were mixed at a 1:1 ratio and transfected with the single-guide RNA construct into Raji cells using the Neon Transfection System (Thermo Fisher Scientific). Single cells were sorted 48 h later into 96-well plates and allowed to expand for 2 wk. Genomic DNA of each single-cell clone was isolated using the Animal Tissue Direct PCR kit (YEASEN; Cat #10180ES70), and the presence of the variant was confirmed by Sanger sequencing.

### Flow cytometry

#### Cell surface flow cytometry staining

Cells were washed with FACS buffer (PBS containing 2% FBS). Fc receptors were blocked by preincubating cells with TrueStain Fc X, and cell viability determination was performed using LIVE/DEAD fixable stains. Cells were stained with antibody cocktails for 30 min at 4°C, after which cells were washed twice with FACS buffer and analyzed by the BD LSR Fortessa cell analyzer.

#### Intracellular flow cytometry staining

Cells were fixed with 1.6% paraformaldehyde (PFA; Thermo Fisher Scientific; Cat #28908) for 20 min at room temperature after surface staining. Fixed cells were washed and permeabilized with intracellular stain perm buffer (Invitrogen; Cat #00-5523-00). Cells were intracellularly stained with antibody cocktails in Perm buffer for 30 min at room temperature and washed twice with FACS buffer before detection.

#### Phospho-flow staining

Cell surface staining was performed as indicated above. In some experiments, cells were treated with CXCL12 at 200 ng/ml (Peprotech; Cat #30028A) and carrier (DMSO) or GGG (100 nM) for 5 min at 37°C. Treated or nontreated cells were immediately fixed with prewarmed 1.6% PFA for 10 min at 37°C. Cells were permeabilized with PERM III buffer (BD; Cat #558050) for 30 min on ice, after which cells were stained with antibody cocktails for 40 min at room temperature. Cells were washed twice with FACS buffer and analyzed by the BD LSRFortessa cell analyzer.

Detailed information on antibodies is provided in Table S2.

### Protein degradation assay

Retrovirally transduced NIH/3T3 cells or Raji CRISPR cells were exposed to CHX (100 µg/ml; Sigma-Aldrich; Cat #C1988) for indicated times. Cells were fixed in 1.6% PFA (Thermo Fisher Scientific; Cat #28908) for 20 min at room temperature. Total P2RY8 expression was analyzed by flow cytometry. The amount of P2RY8 protein remaining at different time points was calculated as a percentage of P2RY8 expression at time 0.

### SRF-luciferase assay

HEK293 cells were transfected with an SRF Luciferase Reporter Plasmid (Yeasen; Cat #11584ES03), pRL-CMV Renilla luciferase control reporter, and indicated vectors. Firefly and Renilla luciferase activities were detected using Dual-Glo luciferase assay kits (Promega; Cat #E1960) according to the manufacturer’s instructions. Results are expressed as ratios of firefly luciferase activity to Renilla luciferase activity.

### Transwell migration assay

#### Transwell migration assay on murine B cells

Retrovirally transduced splenic B cells were washed twice and resuspended with migration media (Gibco RPMI 1640 supplemented with 0.5% fatty acid–free BSA, 1% penicillin/streptomycin, and 1% Hepes) at 10^7^ cells/ml. Migration medium containing CXCL12 (Peprotech; Cat #30028A) and either DMSO (carrier) or 50 nM GGG was then prepared and added into a 24-well plate with 600-µl well volume. Transwell insert (Corning; Cat #CLS3421) was placed on top of each well. 100 µl of cells was added into the insert and allowed to migrate for 3 h, after which migrated cells were collected and analyzed by flow cytometry.

#### Transwell migration assay on human PBMCs

Thawed PBMCs were washed twice and resuspended with migration media at 5 × 10^6^ cells/ml. 100 µl of cells (5 × 10^5^ cells) was added to the Transwell insert and allowed to migrate toward CXCL12 with 100 nM GGG or DMSO control for 3 h, after which cells in the bottom well were collected and analyzed by flow cytometry.

### Transduced splenic B cell localization

24 h after the second spinfection, transduced polyclonal B cells were collected from each 6-well plate and washed twice. Approximately 6 × 10^7^ empty vector–GFP, WT P2RY8–GFP, or mutant P2RY8-GFP was adoptively transferred into immunized adult C57BL6/J (B6/J) mice on day 6 after i.p. immunization with sheep RBCs (SRBCs; Colorado Serum Company; Cat #38112). Mice were analyzed 24 h after transfer. Transduced cells comprised 1–3% (GFP) of all B cells in the spleen by flow cytometry. Positioning of GFP-expressing B cells was tracked by immunofluorescence.

### PBMC stimulation assay

Thawed PBMCs were washed twice and resuspended in RPMI 1640 medium containing 10% FBS, 10 mM Hepes, 55 µM β-mercaptoethanol, 2 mM glutamine, and 50 IU penicillin/streptomycin.  Cells were plated at 3×10^5^ cells/96-well and stimulated with IL-6 (20 ng/ml; Peprotech; Cat #200-06), IL-2 (100 U/ml; Peprotech; Cat #200-02), IL-21 (20 ng/ml; Peprotech; Cat #200–21), ETP (50 µM; MedChemExpress; Cat #HY13629), IFN-α2 (50 ng/ml; BioLegend; Cat #592702), IFN-β (50 ng/ml; Peprotech; Cat #200-06), IFN-γ (20 ng/ml; Peprotech; Cat #300-02), LPS (1 µg/ml; Sigma-Aldrich; Cat #L6529), αIgA, G, M (5 µg/ml, Jackson ImmunoResearch; Cat #109-006-064), GGG (100 nM), IL-10 (50 ng/ml; Peprotech; Cat #200-10), R837 (5 µg/ml; InvivoGen; Cat #tlrl-imqs), or CPG-A (0.5 µM; InvivoGen; Cat #tlrl-2216). P2RY8 expression was analyzed by flow cytometry after 24 h or 48 h of stimulation.

### Immunofluorescence

For F-actin analysis, 2 × 10^4^ NIH/3T3 cells were plated into a Millicell EZ SLIDE 8-well glass (Merck; Cat #PEZGS0816) 18 h before staining. Cells were fixed in 4% PFA (Thermo Fisher Scientific; Cat #28908) at room temperature for 20 min and permeabilized by 0.1% Triton X-100 for 5 min. Cells were stained for F-actin using tetramethylrhomadine-phalloidin (Yeasen; Cat #40734ES75) for 30 min, after which nucleic acid was stained using Hoechst 33258 (Yeasen; Cat #40730ES03). Images were obtained using a NIKON ECLIPSE Ti microscope and analyzed through ImageJ software. Quantification was performed as (1) convert single-channel color images to grayscale before proceeding and (2) adjust the threshold, limit the measured area to just the object (Red), and measure the intensity.

To track the positioning of GFP-expressing B cells, mouse spleens were fixed in 4% PFA for 2 h at 4°C, washed with PBS, submerged in 30% sucrose overnight, and embedded in optimal cutting temperature. Cryosections of 7 µm were dried for 1 h at room temperature and rehydrated in PBS containing 0.1% fatty acid–free BSA for 10 min. A solution consisting of 1% normal mouse serum and 1:100 AF488-conjugated rabbit anti-GFP (Invitrogen; Cat #A21311), 1:100 biotin-conjugated anti-mouse CD35 (BD Bioscience; Cat # 553816), and 1:100 AF647-conjugated anti-mouse IgD (BioLegend; Cat #405708) was used to label transduced B cells, follicular dendrite cells (FDC)s, and endogenous naive B cells, respectively, and was incubated with the slides overnight at 4°C. The slides were then washed in PBS and stained with AF555-conjugated streptavidin (Life Technologies; Cat #S-21381) for 1 h at room temperature, and images were captured with a Zeiss AxioObserver Z1 inverted microscope. For quantification, tiled immunofluorescence spleen images were imported into IMARIS software (v9.6). Using the “surface” function, single B cell follicles were chosen as the region of interest. GFP^+^ cells within these follicles were automatically labeled by the software using the “spot” function. The central region of the follicle was defined with a second surface function, based on CR1 staining of the FDC network. Using the “Filter” function, GFP^+^ cells (spots) in the follicle that were located within the central region of the follicle, more than 6,000 AU (as defined by IMARIS) from the outer border of the FDC network, were filtered out. The remaining spots, defined by their location in the periphery of the follicle, were quantified, and a ratio of peripheral follicular GFP^+^ cells to total follicular GFP^+^ cells was graphed. All follicles with an area larger than 2e9 AU^2^ were quantified for three 10× tiled spleen images per condition.

### Mice and bone marrow chimeras

All mice were housed in a specific pathogen–free facility in the Laboratory Animal Research Center, and all experiments conformed to ethical principles and guidelines that were approved by the University of California, San Francisco Institutional Animal Care and Use Committee or by Renji Hospital Ethics Committee of Shanghai Jiaotong University School of Medicine. Littermate controls were used for experiments, mice were allocated to control and experimental groups randomly, sample sizes were chosen based on previous experience to obtain reproducible results, and the investigators were not blinded.

Site-directed–V_H_3H9 heavy chain (HC) Tg mice have been previously described (Chen et al., 1995) and were generously shared by J. Zikherman, University of California, San Francisco, San Francisco, CA. C57BL/6 mice were obtained from The Jackson Laboratories or from Shanghai Laboratory Animal Co., Ltd. (Shanghai, China), and CD45.1^+^ B6 mice were from National Cancer Institute at Charles River. Mice to be used as bone marrow donors (either C57BL/6 or V_H_3H9 HC Tg mice) were injected intravenously with 3 mg 5-fluorouracil (Sigma-Aldrich; Cat #56627). Bone marrow was collected after 4 d and cultured in DMEM containing 15% (vol/vol) FBS, antibiotics (penicillin [50 IU ml^−1^], and streptomycin [50 mg ml^−1^]; Cellgro), and 10 mM Hepes, pH 7.2 (Cellgro) supplemented with IL-3, IL-6, and stem cell factor (at concentrations of 20, 50, or 100 ng ml^−1^, respectively; Peprotech). Cells were spin-infected twice at days 1 and 2 with viral supernatant and transferred into irradiated CD45.1^+^ B6 recipient mice on day 3. Lethally irradiated mice receiving transduced C57BL/6 bone marrow were immunized 8–12 wk after reconstitution, on day 0 and day 7 with SRBC i.p., and then the tissue was harvested on day 10. Immune cell population chimerism was determined by flow cytometry for gates containing at least 50 events. Tissues from mice receiving transduced V_H_3H9 HC bone marrow were harvested at 9–12 wk following reconstitution, with splenic developmental B cell subsets and Vλ, Vλ1, and Vκ usage determined by flow cytometry.

### Mass spectrometry

GGG was measured in the supernatant of cell cultures using the previously described liquid chromatography–tandem mass spectrometry (LC-MS/MS) protocol (Lu et al., 2019). Briefly, the supernatants of polyclonal B cell cultures or NIH/3T3 cell cultures were collected and centrifuged to remove cells and debris. 3M HCl was added to a pH of 3.5, and the supernatants were frozen in liquid nitrogen until time of processing. Upon thawing, 100% methanol was added to form a 20% methanol solution, and 10 nM of the internal standard LTC4-d5 was added. The resulting solution was bound to a 10-g C18 SPE column (Agilent) using a vacuum manifold. Columns were washed with water and 50% methanol, and compounds were then eluted with 100% methanol. The methanol eluent was dried and reconstituted in 200 µl of 100% methanol for LC-MS analysis.

For plasma samples, a modified GGG extraction method was used. 200 µl of plasma was extracted with 1 ml ice-cold 100% MeOH. Samples were vortexed for 1 min, incubated on ice for 30 min, and centrifuged at 4°C at 21,130 rcf. 500 μl of the centrifuged extract was transferred to a glass vial and dried under nitrogen and vacuum. Samples were redissolved in 200 µl of 100% methanol for LC-MS analysis.

For supernatant GGG measurements, a Shimadzu Nexera X2 HPLC coupled to an AB SCIEX QTRAP 6500 mass spectrometer was used for LC-MS analysis. For plasma GGG measurements, a Sciex ExionLC ultra performance liquid chromatography coupled to a SCIEX QTRAP 7500 mass spectrometer was used for LC-MS analysis. Samples were injected (10 µl supernatant, 5 µl plasma) onto a Synergi Polar-RP column (75 × 4.6 mm) and a mobile phase gradient consisting of A: 100% H_2_O + 0.1% formic acid; and B: 100% acetonitrile + 0.1% formic acid. 0–1 min, 40% B; 1–4 min, ramp to 95% B; 4–6 min, 95% B; 6–6.5 min, ramp to 50% B; 6.5–8 min, 40% B was used for analysis. GGG was detected in positive ionization mode using multiple-reaction monitoring scans with ion pair 580.3/179.0. The internal standard, LTC4-d5, was identified with ion pair 631.4/179.0. For the 6500/(7500), the turbo spray ion source was maintained at 400°C, 20 curtain gas (CUR)/(35 CUR), 40 ion source gas 1 and 40 ion source gase 2, 5,500 ion source voltage, with compound settings declustering potential 160, entrance potential 10, and collision cell exit potential 10. A collision energy of 35 eV was used when performing fragmentation analysis. Peak area was integrated using Sciex Analyst software and referenced against a standard curve prepared with synthetic GGG to calculate compound abundance.

### Statistics

Statistical analyses were performed using GraphPad Prism software (version 8,9; GraphPad Software Inc.). Differences between two groups were determined by unpaired Student’s *t *test*,* unpaired Welch’s *t *test, two-way ANOVA, and where indicated, the Mann-Whitney test. Comparisons of several groups were calculated with one-way ANOVA or Kruskal-Wallis test. χ^2^ test was used to test statistical independence. A P value <0.05 was considered statistically significant. Levels of significance were defined as *, P < 0.05; **, P < 0.01; ***, P < 0.001; ****, P < 0.0001.

### Online supplemental materials

Fig. S1 shows identification of L257F variant in patient with SLE. Fig. S2 shows characterization of P2RY8 variants, which indicates loss of function. Fig. S3 shows GGG-mediated inhibition of AKT and ERK activation and cell migration in human PBMCs. Fig. S4 shows that P2RY8 is down-regulated in B cell subsets of SLE patients. Fig. S5 shows that P2RY8 restrains plasma cell formation and enforces B cell negative selection. Table S1 shows basic clinical information of studied patients. Table S2 shows antibodies used in FACS.

## Supplementary Material

Table S1 shows basic clinical information of studied patients.

Table S2 shows the antibodies used in FACS.

## Data Availability

The whole-exome sequences of trio and probands described in the manuscript have been deposited to the Sequence Read Archive database (Accession no. PRJNA770357) or the European Genome-Phenome Archive database under accession no. EGAD00001008330. Source data of this study are available from the corresponding author upon request.
