# Supplementary material for: P2RY8 variants in lupus patients uncover a role for the receptor in immunological tolerance
Source: J Exp Med. 2021 Dec 10;219(1):e20211004. doi: 10.1084/jem.20211004 (PMC8669517; doi:10.1084/jem.20211004)
Supplement: Table S2 — shows the antibodies used in FACS. [file JEM_20211004_TableS2.docx]

Table S2. Antibodies used in FACS

| Antibodies | Source | Identifier |
| --- | --- | --- |
| BV650 anti-Human CD19 | BioLegend | Cat #302238; AB_2562097 |
| BV605 anti-Human CD38 | BioLegend | Cat #303532; AB_2562915 |
| BV711 anti-Human CD24 | BD | Cat #563401; AB_2631261 |
| BV510 anti-Human IgD | BioLegend | Cat #348220; AB_2561945 |
| APC-EF780 anti-Human CD27 | eBioscience | Cat #47-0279; AB_1272040 |
| V450 anti-Human λ light chain | BD | Cat #561379; AB_10681718 |
| BUV395 anti-Human CD11c | BD | Cat #563787; AB_2744274 |
| BV786 anti-Human CD3 | BD | Cat #563799; AB_2744384 |
| BUV496 anti-Human CD4 | BD | Cat #564651; AB_2744422 |
| PE-CF594 anti-Human PD-1 | BioLegend | Cat #329940; AB_2563659 |
| AF647 anti-Human CXCR5 | BD | Cat #558113; AB_2737606 |
| APC-R700 anti-Human CD25 | BD | Cat #565106; AB_2744339 |
| BB700 anti-Human CD127 | BD | Cat #566398; AB_2744279 |
| PE-CY7 anti-Human CD45RA | eBioscience | Cat #25-0458-73; AB_1944378 |
| BUV737 anti-Human CD56 | BD | Cat #564447; AB_2744432 |
| BB700 anti-Human CD127 | BD | Cat #566398; AB_2744279 |
| BV421 anti-Human CXCR4 | BioLegend | Cat #306518; AB_11146018 |
| Anti-Human P2RY8 | ATLAS | Cat #HPA003631; AB_1079543 |
| Rabbit IgG Isotype Control | Invitrogen | Cat #02-6102 |
| Anti-Human Fc block | BioLegend | Cat #422302; AB_2818986 |
| BV605 anti-Mouse CD138 | BD | Cat #563147; AB_2721029 |
| PERCP-CY5.5 anti-Mouse CD45.2 | Tonbo Bio | Cat #65-0454-U100 |
| Pacific Blue anti-Mouse CD21/35 | BioLegend | Cat #123414; AB_2085158 |
| PECY7 anti-Mouse CD23 | BioLegend | Cat #101614; AB_2103036 |
| APC anti-Mouse CD93 | eBioscience | Cat #17-5892-82; AB_469466 |
| Biotin anti-Mouse IgMb | BD | Cat #553519; AB_394900 |
| PE anti-Mouse Ig light chain λ | BioLegend | Cat #407307; AB_1027659 |
| PE anti-Mouse Ig light chain κ | BioLegend | Cat #409505; AB_2563580 |
| Biotin anti-Mouse Ig λ-1 light chain | BD | Cat #553431; AB_394851 |
| Unconjugated anti-Mouse CD16/32 | Bio X Cell | Cat #BE0307; AB_2736987 |
| PE anti-Mouse CD138 | BioLegend | Cat #142504; AB_10916119 |
| BV421 anti-Mouse CD267 (TACI) | BD | Cat #742840; AB_2741091 |
| BV711 anti-Mouse CD98 | BD | Cat #745466; AB_2743009 |
| BV650 anti-Mouse CD45.1 | BioLegend | Cat #110736; AB_2743009 |
| Biotin anti-Mouse CD35 | BD | Cat #553816; AB_395068 |
| AF647 anti-Mouse IgD | BioLegend | Cat #405708; AB_893528 |
| BUV395 anti-Mouse B220 | BD | Cat #563793; AB_2738427 |
| Anti-Mouse Fc Block | BD | Cat #553142; AB_394657 |
| Phospho-Akt (Ser473) (D9E) XP Rabbit | CST | Cat #4060S; AB_2315049 |
| PE anti-ERK1/2 Phospho (Thr202/Tyr204) | BioLegend | Cat #369506; AB_2629705 |
| 488 LIVE/DEAD Stain Kit Green Fluorescent | Invitrogen | Cat #L23101 |
| BV510 LIVE/DEAD Fixable Aqua Dead Cell Stain | Invitrogen | Cat #L34957 |
| APC anti-Human/Mouse CD90.1 (Thy-1.1) | eBioscience | Cat #17-0900-82; AB_469420 |
| BV785 anti-Human/Mouse B220 | BioLegend | Cat #103246; AB_2563256 |
| AF647 anti-rabbit IgG (H+L), F(ab′)2 Fragment | CST | Cat #4414S; AB_10693544 |
| PE Donkey anti-Rabbit IgG | BioLegend | Cat #406421; AB_2563484 |
| PE anti-DYKDDDDK Tag | BioLegend | Cat #637309; AB_2563147 |
| 488 anti-GFP | Thermo Fisher Scientific | Cat #A21311; AB_221477 |
| 488 anti-GFP | BioLegend | Cat #338008; AB_2563288 |
| eFluor 780 anti-Fixable Viability Dye | eBioscience | Cat #65-0865-18 |
| AF555 anti-Streptavidin | Life Technologies | Ca t#S-21381; AB_2307336 |
| BV605 anti-Streptavidin | BioLegend | Cat #405229 |
| TRITC-phalloidin | Yeasen | Cat #40734ES75 |
| iFluor 647 phalloidin | Yeasen | Cat #40762ES75 |
| Hoechst 33258 | Yeasen | Cat #40730ES03 |
